# Comparison of Ketorolac at 3 Doses in Children With Acute Pain: Protocol for A Randomized Controlled Trial

**DOI:** 10.2196/76554

**Published:** 2025-09-26

**Authors:** Mohamed Eltorki, Redjana Carciumaru, Samina Ali, Anne Holbrook, Michael Livingston, Samira Samiee-Zafarghandy, Karen Beattie, Lehana Thabane, Lucia Giglia

**Affiliations:** 1 Department of Pediatrics Cumming School of Medicine University of Calgary Calgary, AB Canada; 2 Department of Pediatrics Faculty of Health Sciences McMaster University Hamilton, ON Canada; 3 Department of Pediatrics Faculty of Medicine and Dentistry University of Alberta Edmonton, AB Canada; 4 Department of Medicine Faculty of Health Sciences McMaster University Hamilton, ON Canada; 5 Department of Surgery Faculty of Health Sciences McMaster University Hamilton, ON Canada; 6 Biostatistical Unit St. Joseph's Research Institute St. Joseph's Hospital Hamilton, ON Canada

**Keywords:** ketorolac, dose-response relationship, dosing, pain management, randomized controlled trial, protocol, emergency department

## Abstract

**Background:**

Intravenous ketorolac is a potent nonopioid analgesic commonly used to treat vigorous pain in children and adults. Despite its widespread use in pediatric emergency settings, ketorolac dosing in children remains “off-label,” with limited high-quality evidence to guide practice. Pharmacokinetic differences between children and adults suggest that lower ceiling doses adopted from adult practice may lead to suboptimal analgesia in pediatric populations. Inconsistent ketorolac dosing practices across centers reflect substantial clinical uncertainty. Amid efforts to reduce opioid use and provide effective nonopioid alternatives, rigorous pediatric trials evaluating ketorolac dosing are urgently needed.

**Objective:**

The primary objective is to determine whether 2 lower-dose intravenous ketorolac strategies (0.25 mg/kg-30 mg or 0.5 mg/kg-10 mg) are noninferior to the standard dosing regimen (0.5 mg/kg-30 mg) in reducing mean pain scores at 60 minutes postadministration in children aged 6-17 years presenting with vigorous pain. The secondary hypothesis is that ketorolac 0.5 mg/kg up to 10 mg will be superior to 0.25 mg/kg up to 30 mg by at least the minimally important difference of 2.0 points on the verbal Numerical Rating Scale (vNRS).

**Methods:**

The KETODOSE trial is a single-center, randomized, double-blind, double-dummy, noninferiority trial conducted at McMaster Children’s Hospital. Eligible participants aged 6 to <18 years with vigorous pain (vNRS >4) are randomized in a 1:1:1 ratio to standard dosing or one of 2 low-dose ketorolac regimens. Study drugs are administered via intravenous push over 5 minutes. Pain scores are assessed at baseline, 30, 60, 90, and 120 minutes. The primary endpoint is the mean change in vNRS score at 60 minutes. Secondary outcomes include pain scores at other time points, time to effective analgesia, rescue analgesia requirements, opioid consumption, and adverse events. Caregiver perceptions regarding analgesic use are evaluated using a mixed-methods semistructured survey. A sample size of 180 participants (60 per group) provides 80% power to detect noninferiority within a margin of 1.0 on the vNRS, assuming an SD of 1.5. Intention-to-treat and per-protocol analyses will be performed.

**Results:**

Recruitment is ongoing. Final analyses will be performed once follow-up is completed for all participants. Results will be disseminated through peer-reviewed publications, conference presentations, caregiver- and clinician-facing educational tools, and national knowledge mobilization networks.

**Conclusions:**

The KETODOSE trial will provide urgently needed evidence to guide ketorolac dosing for acute pain in children. If lower-dose regimens are shown to be noninferior to standard dosing, this may promote safer prescribing practices, reduce adverse events, and support efforts to minimize pediatric opioid use, thereby improving acute pain management in pediatric emergency care.

**Trial Registration:**

ClinicalTrials.gov NCT05641363; https://clinicaltrials.gov/study/NCT05641363

**International Registered Report Identifier (IRRID):**

DERR1-10.2196/76554

## Introduction

### Background

Ketorolac tromethamine is a well-known nonopioid intravenous (IV) analgesic commonly used in children and adults for vigorous pain [1-3]. It is a nonsteroidal anti-inflammatory drug (NSAID) that inhibits the synthesis of inflammatory endogenous substances such as prostaglandins and thromboxanes. Ketorolac has been shown to provide similar analgesia to opioids after outpatient surgical procedures associated with moderate pain, such as fractures, hernia repair, and tonsillectomy [1,4]. Ketorolac is commonly used in the Emergency Department (ED) for renal colic, abdominal pain, chest pain and migraine headaches [5]. However, such use in children is “off-label” as there is a paucity of methodologically sound controlled trials in children to inform optimal and safe dosing.

Previous pharmacological studies show that young children have up to 2- to 4-fold higher clearance and volume of distribution of ketorolac compared to adolescents and adults [6]. This suggests that younger children need a relatively higher dose per kilogram body weight than adults and the currently used 10-15 mg ceiling dose applied to adults may not achieve equivalent analgesia in children [7,8]. Emerging evidence, only applicable to adults, suggests that low-dose ketorolac (10 mg) is as effective as standard dose (30 mg) in acute pain management [5,9-11] with higher doses not conferring further benefit [12]. This has led to clinicians adopting the practice of a 10 mg maximum dose in children without rigorous pediatric evidence. For example, a 10 mg maximum dose in a 50 kg adolescent would equate to 0.2 mg/kg, in contrast to pediatric studies that used 0.5-1 mg/kg, a maximum of 20-30 mg [10,13-16]. In a local survey (February 2022) of our ED physicians, pediatric hospitalists, and surgeons, 10/31 (32%) respondents said that they would administer ketorolac (0.5 mg/kg) up to a maximum of 30 mg, 20/31 (65%) use ketorolac up to a maximum of 10 mg, and 1/31 (3%) did not know the best dose to give. Although the local monograph and order-sets recommend a maximum dose of 30 mg, our local variation in practice is not surprising and reflects clinical uncertainty regarding the optimal dose.

Ketorolac is an excellent alternative to opioids for vigorous pain and it is imperative to conduct pediatric dosing studies to inform both clinicians and regulatory agencies. Given the continued undertreatment of pain in children, its known adverse effects (including prolonged healing times, longer length of stay, less satisfaction) [17], and the lack of high-quality evidence for ketorolac use in children, there is an urgent need for a well-designed, rigorously conducted trial to inform clinical treatment decisions and health care policy. To our knowledge, there are no trials to guide the selection of the most effective and safe ketorolac dose for use in children. This comes at a time when ketorolac, the only available intravenous NSAID in Canada, is being considered increasingly more often to fill the nonopioid gap in treatment for vigorous pain [13,18-21].

### Study Objectives and Hypothesis

#### Primary Objective

In children aged 6-17 years with vigorous pain (as measured on the 11-point verbal Numerical Rating Scale [vNRS]) who are prescribed IV ketorolac by their treating physician, lower-dose IV ketorolac dosing strategies (0.25 mg/kg/dose-30 mg OR 0.5 mg/kg/dose-10 mg) are noninferior to standard dosing (0.5 mg/kg/dose-30 mg) in reducing mean pain scores.

#### Primary Hypothesis

Low-dosing strategies will be noninferior to standard dose by a margin of 1.0 on the 11-point vNRS: (1) ketorolac given at 0.5 mg/kg/dose-10 mg will be noninferior to standard dose ketorolac and (2) ketorolac given at 0.25 mg/kg/dose-30 mg will be noninferior to standard dose ketorolac.

#### Secondary Hypothesis

Dosing at 0.5 mg/kg/dose-10 mg will be superior to 0.25 mg/kg/dose-30 mg by the minimally important difference (MID) of 2.0 on the 11-point vNRS.

#### Secondary Objectives

The objective of this study is to understand patients’ and caregivers’ knowledge, attitudes, perceptions, and emotions on pain, medications, and their use in treating acute pain. As pain is a multidimensional experience, we seek a deeper understanding of participants’ experiences with pain and pain medications by assessing the following domains: (1) caregiver knowledge and attitudes toward widely available analgesics; (2) attitudes and fears associated with IV analgesics; and (3) caregiver perceptions of dosing of analgesics as well as adverse events (AEs) associated with them.

## Methods

### Design

This will be a randomized, noninferiority, double-dummy, quadruple-blinded (participant and their caregiver, clinical staff, research team, outcome assessor, and data analyst), single-center, pediatric ED trial. The study will be conducted using recommendations from the 2010 CONSORT (Consolidated Standards of Reporting Trials) statement extension for noninferiority and equivalence trials [22] (Multimedia Appendix 1).

### Eligibility Criteria

The inclusion and exclusion criteria with their justifications are summarized in Textbox 1.

**Inclusion and exclusion criteria with justification.**

**Inclusion criteria and justification**

1. Age 6 years to <18 years: the primary outcome measure (11-point vNRS) is validated for use in children ≥6 years, and no other evidence-based acute pain measure is recommended for younger children [23-25].
2. Currently experiencing vigorous pain (self-reported pain score >4 using the vNRS at the time of enrollment; ketorolac is used to treat moderate to severe pain) [26].
3. Patients seen in the ED or inpatient setting with acute pain ≤30 days in duration [5].
4. Patient with an IV cannula in situ or ordered (to minimize additional pain or distress).

**Exclusion criteria and justification**

1. Previous enrollment in the trial (to ensure independent observations).
2. Postoperative patients (medically induced pain differs from pathology-related pain).
3. NSAID use within 6 hours or opioid use within 4 hours, or both, before recruitment (to avoid overdosing and confounding).
4. Actively followed and treated by the chronic pain team (response to analgesics may be altered).
5. Cognitive impairment in the caregiver or child (inability to respond to study questions).
6. History of gastrointestinal bleed or ulcers, inflammatory bowel disease, coagulation disorders, cerebrovascular bleeding, or known arterio-vascular malformations (increased bleeding risk).
7. History of chronic and active renal disease (excluding renal calculi and urinary tract infections).
8. History of chronic and active hepatocellular disease (ketorolac is metabolized in the liver).
9. Known pregnancy at the time of enrollment (risk of closure of the patent ductus arteriosus in the fetus).
10. Known hypersensitivity to NSAIDs or opioids.
11. Inability to obtain consent (language barrier without availability of a translator).

### Interventions

To maintain blinding of bedside clinical staff, patients, and research staff, we will administer an active intervention (ketorolac) and 2 controls (identical normal saline placebo) to each patient at 3 differing doses.

1) Standard-dose group A: IV ketorolac 0.5 mg/kg to a maximum of 30 mg, IV normal saline placebo given at 0.25 mg/kg to a maximum of 30 mg, or IV normal saline placebo given at 0.5 mg/kg to a maximum of 10 mg.

2) Low-dose group B. Group B1: IV ketorolac 0.5 mg/kg to a maximum of 10 mg, IV normal saline placebo given at 0.25 mg/kg to a maximum of 30 mg, or IV normal saline placebo given at 0.5 mg/kg to a maximum of 30 mg. Group B2: IV ketorolac 0.25 mg/kg to a maximum of 30 mg, IV normal saline placebo given at 0.5 mg/kg to a maximum of 30 mg, or IV normal saline placebo given at 0.5 mg/kg to a maximum of 10 mg.

For rescue analgesic therapy, any participant can be given any other non-NSAID analgesic at the discretion of the treating physician at least 60 minutes after the trial medication is administered. To minimize overdosing or AEs, any other NSAIDs can be given 6 hours post-intervention.

### Rationale for Treatment Dose

Ketorolac is available at McMaster Children’s Hospital for off-label use in children for vigorous pain. The acceptable dose range is 0.25-1 mg/kg/dose every 6 hours (maximum 30 mg/dose) [27]. Onset of action is 30 minutes after IV administration and the maximum effect is achieved at 2 hours with a total duration of action up to 6 hours. Available population PK study data [28] from 3 studies on 64 children (intraoperative) showed a decrease in both central and peripheral volume of distribution over the first few years of life and clearance decreases from infancy to adolescence. A single dose of 0.5 mg/kg ketorolac (maximum dose 10-30 mg) every 6 hours achieved a half maximal effective concentration at or above the established 0.37 mg/ml. Most of our participants aged 6 years and older will weigh at least 20 kg (50^th^ percentile) and receive the maximum ceiling dose of 10 mg (group B1). Hence, including low-dose group B2 (0.25 mg/kg to a maximum of 30 mg) will allow us to test 2 low dosing strategies since we are changing only one intervention per arm: (1) a lower dose per kg (0.25 mg/kg) is noninferior to standard dose (0.5 mg/kg) and (2) a lower ceiling dose (10 mg) is noninferior to standard ceiling dose (30 mg) (see Multimedia Appendix 2).

### Randomization and Participant Allocation

The study’s research pharmacist at McMaster Children’s Hospital research pharmacy will create a simple computer-generated block randomization list using Robust Randomization App [29] (Center for Biostatistics, Icahn School of Medicine at Mount Sinai) with random varying permutated blocks of 3, 6, and 9 and a 1:1:1 allocation ratio. The research pharmacists will prepare consecutively numbered study drug vials according to the randomization schedule, with clear labels and dosing instructions in each kit. At least 3 study drug kits will be stored in the password-protected ED medication dispensing machines as per standard operating procedures and Division 5 Good Clinical Practice Health Canada Guidelines. Drug kits will be replenished as needed (at a minimum, weekly) by the research pharmacists. Only the research pharmacists will retain the randomization codes.

A log of all screened patients will be maintained. If patients are eligible, details of the study will be discussed with patients and their caregiver. Consent and assent will be obtained as per the Hamilton integrated Research Ethics Board (HiREB) guidelines and approved consenting procedures (see Multimedia Appendix 3 for consent forms). The clinical research assistant will collect baseline clinical variables and will complete the data collection forms. Elements of baseline pain severity (assessed by vNRS), type of pain, location of pain, duration of pain since onset, associated symptoms (eg, nausea, anorexia, vomiting, dysuria, fever, and pain with movement) will be collected. The Clinical Research Assistant will then pick the next consecutively numbered drug kit and enter the drug kit coded number into Research Electronic Data Capture (REDCap; Vanderbilt University). The research nurse will administer the study drugs to the patient (ketorolac and placebo normal saline) according to the dosing guidelines as an IV push over 5 minutes. Five minutes is a safe timeframe to administer IV ketorolac. This is consistent with the instructions on the clinical drug monograph at our hospital. Time 0 will be defined as the point at which administration of the study drug is completed.

### Methods to Protect from Bias

Our study drug kits will contain active ketorolac and normal saline placebo (labelled as ketorolac) with instructions on dosing as per the randomization schedule. Ketorolac is identical to a normal saline placebo in appearance, consistency, and smell. Vials will contain clear liquid ketorolac 30 mg/ml or normal saline placebo ketorolac 30 mg/ml. Vials will be stored in consecutively numbered study drug kits according to the randomization schedule generated by the McMaster Children’s Hospital research pharmacy. The double-dummy design will ensure blinding of the research nurse drawing up and administering the drug. Bedside physicians and nurses, participants, and their families, investigators, research nurses, and clinical research assistants will all be masked to prevent bias in trial procedures and outcome assessment. Once participants are randomized, we will restrict all other pharmacologic analgesic cointerventions except acetaminophen and rescue therapy. Unblinding will not be permitted under any circumstances, as all participants will receive ketorolac and knowledge of the specific dose would not alter clinical management. Other medications that may be part of the patient’s treatment plan, such as antiemetics, antibiotics, or antihistamines, can be coadministered. Because the primary outcome is based on a self-reported pain scale, the semistructured survey will be conducted only after outcome assessments are completed. Research assistants can complete the semistructured survey after the 6-hour outcome assessment or, if the patient is discharged, within 7 days by phone or electronically. The trial is registered on clinicaltrials.gov (NCT05641363) [30].

### Concomitant Medications

To decrease the confounding effects of other analgesics, we will exclude anyone who, before randomization, received any NSAIDs or opioids in the preceding 6 hours and 4 hours, respectively. We will not allow any other concomitant analgesics (except acetaminophen) administration during the study enrollment (from randomization-60 minutes postrescue therapy if it was required). Patients who have taken acetaminophen before randomization will be eligible to participate. Acetaminophen is widely available for use as an over-the-counter medication analgesic and is effective for mild pain but not for moderate or severe pain. Ketorolac should still be effective when given alongside acetaminophen and has a different mechanism of action. In addition, patients with migraine will be allowed to have coadministration of antiemetics such as metoclopramide or ondansetron. This should not affect our outcomes since all those participants will receive ketorolac (at differing doses) as the only analgesic. All cointerventions will be recorded.

### Duration of Treatment and Follow-Up

Ketorolac and a placebo can be given as an IV slow push over 5 minutes with or without a drug pump. Only a single dose will be administered as part of the trial. The effects on pain will be monitored by obtaining baseline pain scores with the vNRS and at times 30, 60, 90, 120 minutes, and at 6 hours if they are still in the hospital. At follow-up, ED and inpatient chart reviews will be completed to determine results of investigations, AEs, total doses of opioids administered in morphine equivalents/kg, all types of NSAIDs administered, dose and frequency for each and the final diagnosis assigned. The survey will be completed within 7 to 14 days once all clinical outcomes are completed (6 hours postintervention).

### Primary and Secondary Outcome Measures

#### Primary Objective Endpoint

Between each low-dose ketorolac group (B1 and B2) and the standard group (A) mean differences in pain as measured on vNRS at 60 minutes. We selected 60 minutes postadministration as the time of our primary outcome assessment after careful consideration and in discussion with our parent and youth collaborators and our clinical pharmacologist coinvestigator. This time point (1) allows children to settle from anxiety associated with IV start, which may confound pain assessment; (2) is an acceptable time point for fair assessment of the analgesic effects of the study drug, with a good balance between onset and peak effect for ketorolac; and (3) is a reasonable timeframe for patients to expect relief from vigorous pain.

#### Secondary Endpoints

Secondary endpoints include the following:

1. vNRS score at 30, 90, and 120 minutes as well as 6 hours post administration.
2. Proportion of participants who achieve the 2-point vNRS MID pain score reduction at 60 and 120 minutes.
3. Proportion of participants who change the severity of their baseline pain category at all time points (mild = 0 to 3, moderate = 4 to 6, severe ≥7 on the vNRS)[24].
4. Time to effective analgesia (ie, time at which a vNRS <3 is achieved postintervention).
5. Proportion of participants requiring any rescue analgesia in each trial arm.
6. Total amount administered as measured by morphine equivalent mg/kg within 8 hours of intervention.

#### Safety Outcomes

Safety outcomes include the following:

1. The proportion of children experiencing any AEs, as reported by caregivers or clinical staff, will be recorded in accordance with the CONSORT Harms checklist [31]. We will solicit specific expected AEs that we found from our SR [32] to be commonly reported with NSAIDs, such as gastric pain. In addition, we will have open-ended questions to collect information on any additional unexpected AEs.
2. Frequency of each specific AE (eg, nausea and vomiting). Time frame is 8 hours post drug administration.

#### Secondary Objective

Semistructured survey with baseline demographics, closed-ended questions (7-point Likert scale), and open-ended questions will be collated, summarized, and analyzed by subgroup (age and gender). Time frame for completion is 7-14 days post randomization.

### Outcome Measurement Tools

We will measure pain using the 11-point vNRS, a validated self-reported acute pain scale for which an anchor-based MID has been established (2 points). The vNRS has demonstrated strong convergent validity, known-groups validity, responsivity, and test-retest reliability in children 6-17 years old with acute pain and in the emergency setting [24,25]. It is rapidly administered by asking, “On a scale of 0 to 10, where 0 means no pain and 10 means the worst pain, how much pain do you have right now?” In a recent systematic review of all acute pain scales for children, the vNRS was the only one to receive a strong recommendation for use in children aged ≥6 years [23]. A phone or electronic questionnaire will be administered after all pain assessments are completed within 7-14 days of enrollment, either in person or by phone or email to assess (1) caregiver knowledge and attitudes toward widely available analgesics; (2) attitudes and fears associated with IV analgesics; and (3) caregiver perceptions of dosing of analgesics as well as AEs associated with them (see Multimedia Appendix 4).

### Sample Size Calculations

Based on adult studies [5,9,11,33], we estimated an SD of 1.5 in each group and a mean difference of 0.2. The noninferiority margin of 1.0 is carefully selected to ensure that a rejection of the null hypothesis would: (1) include the effect size that standard dose ketorolac has historically displayed when compared to placebo (relative risk of achieving 50% pain reduction compared to placebo 2.81, 95% CI [1.8-4.4] with number needed to treat of 2.4, 95% CI [1.8, 3.7]), [10] and (2) exclude clinically important levels of inferiority by keeping our margin at 50% of the known MID of 2.0 on the vNRS, ensuring ethical and relevant clinical application. A statistician (JE) using nQuery Software (Insightful Science), calculated a total sample size of 171 participants (57/group), which would allow us to test our noninferiority hypothesis using a one-sided alpha of 0.025, 80% power, and a noninferiority margin (Δ) of 1.0. Since our primary outcome is short-term (60 minutes) and we are testing a single dose of ketorolac, we expect very few patients withdrawals or loss to follow-up. However, we will increase our sample size by 5%, to 60 per group (total 180 participants), to account for any unanticipated withdrawals.

### Analysis Plan

#### (A) Primary Objective Analysis Plan

Data will be described in terms of mean (SD) or 95% confidence limits for normally distributed data and frequency (percentage) in the case of categorical data. The primary endpoint is the difference between the mean reduction in pain scores between each of the low-dose groups (B1, B2) and the standard-dose group (A) 60 minutes post intervention. The primary analysis will include all participants randomized based on the intention to treat analysis and a sensitivity per-protocol analysis will also be performed. A priori, the primary analysis will be adjusted for baseline pain scores using the analysis of covariance (ANCOVA)*.* Generalized estimating equations will be used to examine the impact of baseline pain score on vNRS outcomes across the entire time frame. Noninferiority would be shown if the lower limit of the 95% CI for the between group (groups B1vs A or B2 vs A) differences in the primary endpoint is >1.0. If >5% [34] of our vNRS scores are missing within 120 minutes, we will use multiple imputation to create 5 datasets wherein any missing pain data will be imputed with the Markov chain Monte Carlo method. For secondary endpoints, we will describe those outcomes using descriptive statistics and perform a linear (continuous), logistic, or ordinal regression (categorical or ordinal). If both low-dose strategies are shown to be noninferior to standard dose, we will conduct an exploratory analysis to see if one low-dosing strategy is superior to the other (group B1 vs B2). This will be conducted using ANCOVA to adjust for baseline pain scores and superiority will be determined by a difference that is defined by the MID of 2.0 on the 11-point vNRS. All analyses will be conducted in a blinded fashion. The research pharmacist will provide the trial statistician with anonymized group allocation codes, without revealing the dosing strategies associated with each group. The group identities will remain blinded to the statistician until the first draft of the manuscript with results has been completed. All analyses will be done using SAS (version 9.4; SAS Inc).

AEs profile analysis: the proportion of children experiencing any AEs as reported by caregivers or health care providers will be compared between groups using logistic regression and presented with their corresponding binomial 95% 2-sided CI. The analysis will evaluate the presence or absence of AEs in each group, such as nausea, epigastric pain, or dyspepsia.

#### (B) Secondary Objective Analysis Plan

Summative statistics will be used to describe the distribution of responses to closed-ended questions. Answers to open-ended questions will be thematically coded; concepts and themes will be developed using a constant comparative method of analysis. Themes related to knowledge of pain and analgesia, fears of AEs or effectiveness of analgesia, satisfaction or dissatisfaction with current therapy will be categorized, among others. Survey findings will not be included in the primary or secondary outcome analyses and will be presented separately as an exploratory component of the study.

### Planned Subgroups

There are 5 planned subgroups:

(1) Underlying painful condition, specifically the 2 most common indications for IV ketorolac, headaches, and acute abdominal pain. No subgroup effect is anticipated given evidence that the cause of pain does not alter response to analgesia [35,36].

(2) Age ≥12 years, since adolescents’ drug metabolism differs from younger age groups. We expect a differential treatment effect but are uncertain regarding direction or magnitude.

(3) Gender (grouped as cisgender, transgender, and other): since gender norms are known to impact a patient’s perceived sensitivity to pain, we will advise participants and caregivers why sex and gender are important in pain research and then use the recommended [37] 2-step questionnaire to determine biological sex and gender separately. The 2-step survey has been tested in transgender populations and validated over a broader North American population [38-42]. We expect a differential treatment effect but are uncertain regarding direction or magnitude.

(4) Sex (male, female, and intersex): since biological mechanisms, such as sex hormones, influence the nervous system’s perception of pain and response to analgesics [43-45]. We expect a differential treatment effect but are uncertain regarding direction or magnitude.

(5) Race and ethnicity: we will collect race and ethnicity data using the standard Canadian Institute for Health Information data questionnaire, with 2 questions on Indigenous identity data standard and race-based data standard [46]. We expect a differential treatment effect but are uncertain regarding direction or magnitude (see Table 1).

**Table 1:** Summary of outcomes and analysis.

Objectives				Outcome measure		Hypothesis		Analysis			
**Primary efficacy outcome^a^**											
		Pain relief		Pain level as reported by participants on an 11-point vNRS^b^ score at 60 minutes post administration of trial drugs.		Ketorolac given at 0.5 mg/kg/dose up to 10 mg or at 0.25 mg/kg/dose up to 30 mg will be noninferior to standard dose ketorolac		Intention to treat analysis using ANCOVA^c^			
**Safety outcome**											
		Group differences in proportion of AEs^d^		The proportion of children experiencing AEs related to study drug administration		Participants who receive low dose will have fewer AEs than those who receive standard dose		Regression analysis			
**Secondary outcomes**											
		**Efficacy**									
			Pain relief at all time points		Between group differences in pain score at all study time points at 30, 90 and120 minutes		Low dose will be noninferior to standard dose at all time points.		GEE^e^: adjusted for baseline pain scores		
			Pain relief by the MID		Proportion who achieves a 2-point vNRS reduction at 60 and 120 minutes		Low dose will be noninferior to standard dose ketorolac regimens in reducing pain by the 2-point MID^f^ on vNRS		Regression analysis		
			Pain category change		Proportion of participants who change their baseline pain category at all time points (mild 1 to 3, moderate 4 to 6, severe ≥7 on vNRS)		No difference between low dose and standard dose ketorolac in pain category change at all time points		Regression analysis		
			Time to achieve mild or no pain level		Time to effective analgesia as measured by the time when vNRS of <3 is achieved		No difference between all ketorolac dosing arms.		Cox regression analysis		
			Rescue analgesia		Proportion of participants requiring any additional analgesia in each trial arm		No difference between all ketorolac dosing arms		Regression analysis		
			Opioid sparring		Total amount of opioid administered within 8 hours of intervention		No difference between all ketorolac dosing arms		Regression analysis		
		**Safety**									
			AEs frequency		Frequency of each specific AE related to study drug administration		There will be more AEs associated with standard dose ketorolac compared to low dose regimens		Regression analysis		
**Subgroups**											
		Underlying condition		Proportion of patients with headaches and acute abdominal pain		No subgroup effect is anticipated given prior evidence [35,36]		Regression analysis			
		Sex assigned at birth		Proportion of male, female, and intersex		Differential treatment effect		Regression analysis			
		Gender		Proportions of genders (as defined by participant)		Differential treatment effect for girl or woman compared to boy or man gender.		Regression analysis			
		Age		Dichotomized to 6-11 years vs 12-17 years		Differential treatment effect based on age		Regression analysis			
		Race and ethnicity		Proportion of participants in each race and ethnicity category		Differential treatment effect based on race and ethnicity		Regression analysis			
**Sensitivity analysis**											
		Per-protocol analysis		Completed for the Primary and Safety outcomes		The primary intention to treat analysis results will remain robust		ANCOVA Regression analysis			

^a^Sample size calculation is based on the primary efficacy outcome.

^b^vNRS: 11-point verbal Numerical Rating Scale.

^c^ANCOVA: analysis of covariance

^d^AEs: adverse events.

^e^GEE: generalized estimating equations.

^f^MID: minimal important difference.

### Frequency of Analysis

The Data Safety Monitoring Board (DSMB) will be provided with a masked comparison between treatment groups with respect to the AEs at the intervals of their choosing. We will inform the DSMB of all related and unexpected serious adverse events within 7 days of their occurrence. At the DSMB’s request, they can receive the associated *P* values for differences in AEs per group. They can further request unmasking. The DSMB board will be completely independent from the trial steering committee and will have a clinical ED physician, a clinical researcher, and a biostatistician who are not associated with or trial sponsor, principal investigators, or had any input on study methodology.

### Patient and Caregiver Advisor Involvement

Patient and caregiver engagement has been integrated throughout the design and conduct of the KETODOSE trial. In alignment with the Canadian Institute of Health Research strategy, we collaborated with a parent advisor (KB) and a youth advisor (MN) from the early stages of protocol development. Our youth and parent advisor provided feedback on the clarity and tone of the survey questions to ensure they were age-appropriate and understandable for caregivers, children, and adolescent participants.

During protocol development, the original plan was to assess the primary outcome (pain reduction) at 2 hours postdrug administration. However, following feedback from both the youth and parent advisors, who emphasized the importance of capturing earlier, meaningful pain relief in the ED context, the primary outcome time point was changed to 60 minutes. This adjustment reflects their input that fast-acting analgesia is a priority for patients and families in acute care settings. Ongoing engagement is planned during the dissemination phase, where our advisors will be invited to codevelop plain-language summaries and caregiver-facing educational materials based on the trial results.

### Trial Day-to-Day Operations

A study coordinator with experience in Health Canada drug trials will work with principal investigators (ME and LG). Clinical Research Assistants and research nurses will be available to recruit from 8 AM to 9 PM. We will present the study to staff physicians in the Pediatric ED, General Pediatrics, and Pediatric Surgery so they are aware of the study eligibility criteria and how to contact the study team members. Our research nurse will perform daily rounds in the ED and on the wards during clinical rounds to ask and remind the clinical team of any potential participants. The clinical team will ask potential participants if they would like to be approached by research personnel since they may be eligible for the KETODOSE trial. If the patients agree, they will be approached by research staff to check eligibility. Eligibility will then be confirmed with the treating physician or principal investigators, or both. If confirmed, the details of the study will be discussed with the patient and caregiver.

Children and caregivers will be consented and assented for participation in the trial, including the follow-up survey. Once caregivers’ consent and participants’ assent, the research nurse will obtain the drug kit from the ED drug dispensing machine and assign them the drug kit with the smallest subsequent number. Data will be prospectively collected on baseline characteristics, including age, sex, gender, baseline pain severity (vNRS), type of pain, location, and duration of pain and associated symptoms. Following randomization, the study drug kit containing the study drug vials with information on dosing will be administered to the patient by the research nurse or bedside nurse with the clinical research assistant’s help. Each patient will receive a single dose of IV ketorolac and 2 doses of normal saline as outlined in the intervention section above. Time 0 (baseline) will be considered the time at which study drug administration has been completed. Pain assessments will be conducted at 30-, 60-, 90-, and 120-minute intervals following study drug administration. A chart review will be performed approximately one week after the index visit to identify any return visits to the ED and document the reasons for representation, as well as abstract all data regarding other analgesia administered during the enrollment visit.

### Safety Oversight

Safety oversight will be under the direction of a DSMB composed of individuals with the appropriate expertise, including trial methodology, epidemiology and biostatistics, and a pediatrician or a pediatric surgeon. Members of the DSMB will be independent from the study conduct and free of conflict of interest, or measures should be in place to minimize perceived conflict of interest. The DSMB will meet at least semiannually to assess safety and efficacy data for both trials in this study. The DMSB will operate under the rules of an approved charter terms of reference, that will be reviewed at the organizational meeting of the DSMB. At this time, each data element that the DSMB needs to assess will be defined. The DSMB will provide its input to the principal investigators and the Study coordinator. The DSMB may also provide input to the HiREB and Health Canada if requested.

### Trial Monitoring

Trial conduct will be actively monitored by the study team at McMaster Children’s Hospital. A study coordinator will oversee daily screening, enrollment, data collection, and protocol adherence. Additional monitoring procedures were completed by Hamilton Health Sciences Research Office, including review of REDCap data entry, verification of informed consent, compliance with dosing and outcome measurement protocols, and documentation of any protocol deviations or AEs. Study drug kits will be tracked, and adherence logs will be maintained. Weekly reviews of trial operations are conducted by the principal investigators and study coordinator, and drug accountability will be overseen by the research pharmacy.

### Ethical Considerations

This trial was reviewed and approved by the HiREB, affiliated with McMaster University and Hamilton Health Sciences (see Multimedia Appendix 5). The study adheres to all ethical standards for research involving human participants, as outlined in the Tri-Council Policy Statement: Ethical Conduct for Research Involving Humans, and the principles of the Declaration of Helsinki. Written informed consent was obtained from all participants’ parents or legal guardians before enrollment, and assent was obtained from children aged 7 years and older. Participants were informed of their right to withdraw at any time without any impact on their clinical care. Privacy and confidentiality were strictly maintained throughout the study. All participant data were deidentified before analysis and stored securely in a password-protected local database (REDCap) [47] stored at Hamilton Health Sciences, Population Health Research Institute. Only authorized study team members had access to identifiable data. Identifying information was stored separately from study data in compliance with institutional data privacy standards. As this trial involved children, a potentially vulnerable population, special care was taken to ensure that consent was freely given, and that families had sufficient time and opportunity to discuss participation with an adult substitute decision maker before deciding to enroll. In addition, mature minor consent was used in this trial and capacity to consent was assessed by the research team and assented by the caregiver or substitute decision maker. Participants who completed the follow-up survey received a CAD $5 (US $3.7) gift card as a token of appreciation for their time. No other financial incentives were provided for study participation.

## Results

The KETODOSE trial received funding in June 2022 from the Core Builder’s Grant, Department of Pediatrics, McMaster University. We received ethics approval from the Hamilton Integrated Research Ethics Board in April 2023 and a Health Canada No Objection Letter in January 2023 (see Multimedia Appendix 6). Our first enrollment was on May 23, 2023, at McMaster Children’s Hospital, Hamilton Health Sciences. At the time of this protocol submission, from July 2023 to April 2025, we have enrolled 170 participants. We expect to complete recruitment by June 2025, with data analysis commencing in July 2025. Trial results are expected to be submitted for publication in Winter 2026.

The main challenge we encountered was that many participants were excluded due to having received NSAIDs within 6 hours before recruitment, since many participants received ibuprofen prior to recruitment. While shortening that dosing gap to 3-hour would increase recruitment, we felt that a 6-hour washout period between NSAID doses is important to ensure that our intervention effect is not confounded by a recent NSAID treatment. This decision was discussed in the interim DSMB safety and futility analysis, and it was suggested to continue the trial without modification since the trial was feasible and progressing well.

## Discussion

The KETODOSE trial addresses a critical evidence gap in determining optimal ketorolac dosing for common acute pain indications in the ED, where current practices have relied largely on adult data and expert opinion. Should our findings demonstrate that lower-dose regimens (0.25 mg/kg up to 30 mg and 0.5 mg/kg up to 10 mg) are noninferior to standard dosing, this would provide the first high-quality evidence to support dose de-escalation in children with acute pain. Such findings could validate the common, but unproven, practice of using a 10 mg ceiling dose in children. Moreover, this trial tests whether ceiling dose or per-kilogram dosing is the driver of analgesic effectiveness. If 0.5 mg/kg up to a maximum of 10 mg is found to be noninferior to the standard 0.5 mg/kg up to 30 mg regimen, yet superior to 0.25 mg/kg up to 30 mg by the MID, this would suggest that a higher per-kilogram dose combined with a lower ceiling dose may represent the optimal ketorolac strategy for children, balancing efficacy with reduced drug exposure.

In preparation for this trial, we conducted and published [48] a systematic review and meta-analysis of 4 databases and trial registries in early 2022 to identify any RCTs regarding ketorolac dosing. We identified four RCTs that included 392 adult participants. Studies compared standard dose ketorolac (30 mg) to either mid-dose ketorolac (15-20 mg) or low-dose ketorolac (10 mg). Most trials had small sample sizes and used a single dose of IV ketorolac [5,9,11], with the exception of one study that used intramuscular ketorolac.[33] All trials used the 100-mm visual analogue scale to measure their primary outcome pain level at 1 hour (3 trials) or 4 hours (1 trial) [11] postintervention. Four trials with 196 participants compared IV ketorolac 30 mg to 15-20 mg, with a nonsignificant mean difference of 1.03 mm (95% CI –2.45 to 4.5). Two trials with 116 participants compared ketorolac 30 mg to 10 mg and found a nonsignificant mean difference of 1.55 mm (95% CI –2.42 to 5.53) favoring the 30 mg dose. There was consistency across all trials and an *I*^2^ of 0%. This suggests that, for adults with acute pain, there is no clinically or statistically meaningful difference between lower doses of ketorolac and standard doses, and that an analgesic ceiling to ketorolac is reached at 10 mg in adults. However, there were no published pediatric trials or registered ongoing trials on trial registries.

The KETODOSE trial has several key strengths. First, it is the first randomized, double-dummy, quadruple-blind trial to rigorously evaluate different IV ketorolac dosing strategies in a pediatric population presenting with acute pain. Second, the trial is designed with a clinically meaningful and conservative noninferiority margin of 1.0 on the 11-point vNRS, representing 50% of the established MID. This tight margin ensures that any declaration of noninferiority will reflect truly comparable clinical effectiveness, thereby supporting safe and ethically sound dose reduction decisions. Third, the primary outcome time point at 60 minutes was carefully selected in collaboration with caregivers and pharmacologic experts to balance drug onset with clinical practicality, enhancing the relevance of results to ED settings. The inclusion of multiple secondary outcomes, such as time to effective analgesia, opioid sparing, and AEs, further strengthens the clinical applicability. Fourth, the mixed-methods component, capturing caregiver perceptions and attitudes toward analgesics, offers insights into the patient-family experience, which is critical for future implementation and guideline uptake.

Despite these strengths, several limitations merit consideration. As a single-center trial, generalizability to other settings may be limited, particularly where baseline analgesic practices or patient populations differ. Our strict exclusion of children who had recently received NSAIDs, while necessary to avoid confounding, reduced enrollment and may affect the applicability of findings to real-world scenarios where pre-hospital ibuprofen use is common. Nonetheless, the decision to maintain a 6-hour NSAID washout was supported by our DSMB and prioritized internal validity.

We will disseminate trial findings through peer-reviewed publications, presentations at national pediatric and emergency medicine conferences, and targeted knowledge translation products such as evidence-based dosing tools and caregiver-facing educational materials. We also aim to collaborate with national pediatric emergency networks to support widespread adoption and inform future studies comparing ketorolac with other nonopioid analgesics. Ultimately, this trial will contribute urgently needed data to optimize ketorolac use in children, with the potential to improve pain management outcomes while promoting safer prescribing for children with acute pain.

### Conclusions

The KETODOSE trial will provide rigorous evidence comparing standard and low-dosing strategies for IV ketorolac in children with an acute painful condition, seen and treated in the ED. Using a randomized, double-dummy, quadruple-blind, noninferiority design with a conservative margin anchored to the validated MID, this study directly tests whether dose de-escalation strategies (0.25 mg/kg up to 30 mg; 0.5 mg/kg up to 10 mg) achieve analgesia comparable to the current standard (0.5 mg/kg up to 30 mg). If noninferiority is demonstrated, findings will support safer prescribing with lower drug exposure, reduce unwarranted practice variation, and inform ED protocols, caregiver education, and future multicenter studies comparing ketorolac with other analgesics. Regardless of outcome, the results will fill a critical evidence gap and guide clinicians, policymakers, and regulators toward more consistent, evidence-based pain management for children.

## Appendix

Multimedia Appendix 1 SPIRIT checklist.

Multimedia Appendix 2 Ketorolac tromethamine product monograph.

Multimedia Appendix 3 Consent forms.

Multimedia Appendix 4 Patient and caregiver survey.

Multimedia Appendix 5 Ethics certificate.

Multimedia Appendix 6 Health Canada no objection letter.

Multimedia Appendix 7 Peer review report by the Core Builder Team Grant Committee, Department of Pediatrics at McMaster University (Canada).

## Abbreviations

AE: adverse event
ANCOVA: analysis of covariance
CONSORT: Consolidated Standards of Reporting Trials
DSMB: Data Safety Monitoring Board
ED: emergency department
GEE: generalized estimating equations
HiREB: Hamilton integrated Research Ethics Board
IV: intravenous
MID: minimally important difference
NSAID: nonsteroidal anti-inflammatory drug
RCT: randomized controlled trial
REDCap: Research Electronic Data Capture
vNRS: verbal Numerical Rating Scale

## Data Availability

The datasets generated or analyzed during this study are available from the corresponding author on reasonable request.
