# Supplementary material for: Comparison of Ketorolac at 3 Doses in Children With Acute Pain: Protocol for A Randomized Controlled Trial
Source: JMIR Res Protoc. 2025 Sep 26;14:e76554. doi: 10.2196/76554 (PMC12550451; doi:10.2196/76554)
Supplement: Multimedia Appendix 2 [file resprot_v14i1e76554_app2.pdf]

PRODUCT MONOGRAPH

Pr Ketorolac Tromethamine Injection, USP

30 mg/mL sterile solution for
intramuscular injection

Non-Steroidal Anti-Inflammatory Drug (NSAID)

Juno Pharmaceuticals Corp.
402-2233 Argentia Rd.
Mississauga, Ontario
L5N 2X7

Date of Revision:
September 21, 2020

Submission Control No: 243158

Table of Contents

PART I: HEALTH PROFESSIONAL INFORMATION.. 3
SUMMARY PRODUCT INFORMATION 3
INDICATIONS AND CLINICAL USE 3
CONTRAINDICATIONS 4
WARNINGS AND PRECAUTIONS 5
ADVERSE REACTIONS 15
DRUG INTERACTIONS…………………………………………………………………..17
DOSAGE AND ADMINISTRATION 19
OVERDOSAGE 21
ACTION AND CLINICAL PHARMACOLOGY 21
STORAGE AND STABILITY……………………………………………………………..24
SPECIAL HANDLING INSTRUCTIONS……………………………………………... 24
DOSAGE FORMS, COMPOSITION AND PACKAGING 25

PART II: SCIENTIFIC INFORMATION.. 26
PHARMACEUTICAL INFORMATION 26
DETAILED PHARMACOLOGY 26
TOXICOLOGY 29
REFERENCES 37

PART III: CONSUMER INFORMATION... 39

Pr KETOROLAC TROMETHAMINE INJECTION, USP

ketorolac tromethamine

PART I: HEALTH PROFESSIONAL INFORMATION

SUMMARY PRODUCT INFORMATION

Route of Administration	Dosage Form / Strength	Non-medicinal Ingredients
Intramuscular	30 mg/mL sterile solution for injection	10% (w/v) alcohol, sodium chloride, sodium hydroxide, hydrochloric acid.

INDICATIONS AND CLINICAL USE

Intramuscular Route:

Intramuscular injection of Ketorolac Tromethamine Injection, USP (ketorolac tromethamine) is indicated for:

- the short-term management (not to exceed 2 days) of moderate to severe acute pain, including pain following major abdominal, orthopaedic and gynaecological operative procedures.

The total duration of combined intramuscular and oral treatment should not exceed 5 days.

For patients with an increased risk of developing CV and/or GI adverse events, other management strategies that do NOT include the use of NSAIDs should be considered first (see CONTRAINDICATIONS and WARNINGS AND PRECAUTIONS).

Use of ketorolac tromethamine should be limited to the lowest effective dose for the shortest possible duration of treatment in order to minimize the potential risk for cardiovascular or gastrointestinal adverse events (see CONTRAINDICATIONS and WARNINGS AND PRECAUTIONS).

Ketorolac tromethamine, as a NSAID, does NOT treat clinical disease or prevent its progression.

Ketorolac tromethamine, as a NSAID, only relieves symptoms and decreases inflammation for as long as the patient continues to take it.

Geriatrics (> 65 years of age):
Evidence from clinical studies and post-market experience suggests that use in the geriatric population is associated with differences in safety (see WARNINGS AND PRECAUTIONS, Special Populations, Geriatrics).

Pediatrics (< 18 years of age):
Safety and efficacy have not been established in the pediatric population.

CONTRAINDICATIONS

Ketorolac tromethamine is contraindicated in:

- the peri-operative setting of coronary artery bypass graft surgery (CABG). Although ketorolac tromethamine has NOT been studied in this patient population, a selective COX-2 inhibitor NSAID studied in such a setting has led to an increased incidence of cardiovascular/thromboembolic events, deep surgical infections and sternal wound complications
- the third trimester of pregnancy, because of risk of premature closure of the ductus arteriosus and prolonged parturition
- labour and delivery because, through its prostaglandin synthesis inhibitory effect, it may adversely affect fetal circulation and inhibit uterine musculature, thus increasing the risk of uterine haemorrhage
- women who are breastfeeding, because of the potential for serious adverse reactions in nursing infants
- severe uncontrolled heart failure
- known hypersensitivity to ketorolac tromethamine or to other NSAIDs, including any of the components/excipients
- history of asthma, urticaria, or allergic-type reactions after taking ASA or other NSAIDs (i.e. complete or partial syndrome of ASA-intolerance - rhinosinusitis, urticaria/angioedema, nasal polyps, asthma). Fatal anaphylactoid reactions have occurred in such individuals. Individuals with the above medical problems are at risk of a severe reaction even if they have taken NSAIDs in the past without any adverse reaction. The potential for cross-reactivity between different NSAIDs must be kept in mind (see WARNINGS AND PRECAUTIONS: Hypersensitivity Reactions, Anaphylactoid Reactions)
- active gastric / duodenal / peptic ulcer, active GI bleeding
- inflammatory bowel disease
- cerebrovascular bleeding or other bleeding disorders
- coagulation disorders, post-operative patients with high haemorrhagic risk or incomplete haemostasis in patients with suspected or confirmed cerebrovascular bleeding.
- immediately before any major surgery and intraoperatively when haemostasis is critical because of the increased risk of bleeding
- severe liver impairment or active liver disease
- moderate to severe renal impairment (serum creatinine >442 µmol/L and/or creatinine clearance <30 mL/min or 0.5 mL/sec) or deteriorating renal disease (individuals with

lesser degrees of renal impairment are at risk of deterioration of their renal function when prescribed NSAIDs and must be monitored) (see WARNINGS AND PRECAUTIONS: Renal)

- known hyperkalemia (see WARNINGS AND PRECAUTIONS**:** Renal, Fluid and Electrolyte Balance)
- concurrent use with other NSAIDs due to the absence of any evidence demonstrating synergistic benefits and potential for additive side effects
- neuraxial (epidural or intrathecal) administration of Ketorolac Tromethamine Injection due to its alcohol content
- concomitant use with probenecid (see DRUG INTERACTIONS)
- concomitant use with oxpentifylline (see DRUG INTERACTIONS)
- children and adolescents aged less than 18 years

WARNINGS AND PRECAUTIONS

Risk of Cardiovascular (CV) Adverse Events: Ischemic Heart Disease, Cerebrovascular Disease, Congestive Heart Failure (NYHA II-IV) (see WARNINGS AND PRECAUTIONS: Cardiovascular).

Ketorolac tromethamine is a non-steroidal anti-inflammatory drug (NSAID). Use of some NSAIDs is associated with an increased incidence of cardiovascular adverse events (such as myocardial infarction, stroke or thrombotic events) which can be fatal. The risk may increase with duration of use. Patients with cardiovascular disease or risk factors for cardiovascular disease may be at greater risk.

Caution should be exercised in prescribing ketorolac tromethamine to any patient with ischemic heart disease (including but NOT limited to acute myocardial infarction, history of myocardial infarction and/or angina), cerebrovascular disease (including but NOT limited to stroke, cerebrovascular accident, transient ischemic attacks and/or amaurosis fugax) and/or congestive heart failure (NYHA II-IV).

Use of NSAIDs, such as ketorolac tromethamine, can promote sodium retention in a dose-dependent manner, through a renal mechanism, which can result in increased blood pressure and/or exacerbation of congestive heart failure. (see also WARNINGS AND PRECAUTIONS: Renal, Fluid and Electrolyte Balance)

Randomized clinical trials with ketorolac tromethamine have not been designed to detect differences in cardiovascular events in a chronic setting. Therefore, caution should be exercised when prescribing ketorolac tromethamine.

Risk of Gastrointestinal (GI) Adverse Events (see WARNINGS AND PRECAUTIONS: Gastrointestinal)

Use of NSAIDs, such as ketorolac tromethamine, is associated with an increased incidence of gastrointestinal adverse events (such as peptic/duodenal ulceration, perforation, obstruction and gastrointestinal bleeding).

<u>General</u>

The long-term use of ketorolac tromethamine is not recommended as the incidence of side-effects increases with the duration of treatment (see INDICATIONS and DOSAGE AND ADMINISTRATION).

Frail or debilitated patients may tolerate side effects less well and therefore special care should be taken in treating this population. **To minimize the potential risk for an adverse event, the lowest effective dose should be used for the shortest possible duration.** As with other NSAIDs, caution should be used in the treatment of elderly patients who are more likely to be suffering from impaired renal, hepatic or cardiac function. For high risk patients, alternate therapies that do not involve NSAIDs should be considered.

Ketorolac tromethamine is NOT recommended for use with other NSAIDs, with the exception of low-dose ASA for cardiovascular prophylaxis, because of the absence of any evidence demonstrating synergistic benefits and the potential for additive adverse reactions. (See DRUG INTERACTIONS: Drug-Drug Interactions, Acetylsalicylic acid (ASA) or other NSAIDs).

<u>Carcinogenesis and Mutagenesis</u>

(See PART II, TOXICOLOGY: Carcinogenicity, Mutagenicity).

<u>Cardiovascular</u>

Ketorolac tromethamine is a non-steroidal anti-inflammatory drug (NSAID). Use of some NSAIDs is associated with an increased incidence of cardiovascular adverse events (such as myocardial infarction, stroke or thrombotic events) which can be fatal. The risk may increase with duration of use. Patients with cardiovascular disease or risk factors for cardiovascular disease may be at greater risk.

Caution should be exercised in prescribing ketorolac tromethamine to patients with risk

factors for cardiovascular disease, cerebrovascular disease or renal disease, such as any of the following (NOT an exhaustive list)

- **Hypertension**
- **Dyslipidemia / Hyperlipidemia Diabetes**
- **Mellitus**
- **Congestive Heart Failure (NYHA I) Coronary**
- **Artery Disease (Atherosclerosis) Peripheral**
- **Arterial Disease**
- **Smoking**
- **Creatinine Clearance < 60 mL/min or 1 mL/sec**

Use of NSAIDs, such as ketorolac tromethamine, can lead to new hypertension or can worsen pre-existing hypertension, either of which may increase the risk of cardiovascular events as described above. Thus blood pressure should be monitored regularly. Consideration should be given to discontinuing ketorolac tromethamine should hypertension either develop or worsen with its use.

Use of NSAIDs, such as ketorolac tromethamine, can induce fluid retention and edema, and may exacerbate congestive heart failure, through a renally-mediated mechanism (see WARNINGS AND PRECAUTIONS: Renal, Fluid and Electrolyte Balance).

For patients with a high risk of developing an adverse CV event, other management strategies that do NOT include the use of NSAIDs should be considered first. **To minimize the potential risk for an adverse CV event, the lowest effective dose should be used for the shortest possible duration.**

Endocrine and Metabolism

Corticosteroids: Ketorolac tromethamine is NOT a substitute for corticosteroids. It does not treat corticosteroid insufficiency. Abrupt discontinuation of corticosteroids may lead to exacerbation of corticosteroid-responsive illness. Patients on prolonged corticosteroid therapy should have their therapy tapered slowly if a decision is made to discontinue corticosteroids (see DRUG INTERACTIONS: Drug-Drug Interactions, Glucocorticoids).

Gastrointestinal

Serious GI toxicity (sometimes fatal), such as peptic/duodenal ulceration, inflammation, perforation, obstruction and gastrointestinal bleeding, can occur at any time, with or without warning symptoms, in patients treated with NSAIDs, such as ketorolac tromethamine. Minor upper GI problems, such as dyspepsia, commonly occur at any time. Health care providers should remain alert for ulceration and bleeding in patients treated with ketorolac tromethamine, even in the absence of previous GI tract symptoms. Most spontaneous reports of fatal GI events are in elderly or debilitated patients and therefore special care should be taken in treating this population. The incidence of these complications increases with increasing dose. **To minimize the potential risk for an adverse GI event, the lowest effective dose should be used for the shortest possible duration.** For high risk patients, alternate therapies that do not involve NSAIDs should be considered (see WARNINGS AND PRECAUTIONS: Special Populations, Geriatrics).

Patients should be informed about the signs and/or symptoms of serious GI toxicity and instructed to discontinue using ketorolac tromethamine and seek emergency medical attention if they experience any such symptoms. The utility of periodic laboratory monitoring has NOT been demonstrated, nor has it been adequately assessed. Most patients who develop a serious upper GI adverse event on NSAID therapy have no symptoms. Upper GI ulcers, gross bleeding or perforation, caused by NSAIDs, appear to occur in approximately 1% of patients treated for 3-6 months, and in about 2-4% of patients treated for one year. These trends continue, thus increasing the likelihood of developing a serious GI event at some time during the course of therapy. Even short-term therapy has its risks.

Caution should be taken if prescribing ketorolac tromethamine to patients with a prior history of peptic/duodenal ulcer disease or gastrointestinal bleeding as these individuals have a greater than 10-fold higher risk for developing a GI bleed when taking a NSAID than patients with neither of these risk factors.

Other risk factors for GI ulceration and bleeding include the following: *Helicobacter pylori* infection, increased age, prolonged use of NSAID therapy, excess alcohol intake, smoking, poor general health status or concomitant therapy with any of the following:

- Anti-coagulants (e.g. warfarin)
- Anti-platelet agents (e.g. ASA, clopidogrel) Oral
- corticosteroids (e.g. prednisone)
- Selective Serotonin Reuptake Inhibitors (SSRIs) (e.g. citalopram, fluoxetine, paroxetine, sertraline)

Close medical supervision is recommended in patients prone to gastrointestinal tract irritation. In these cases, the physician must weigh the benefits of treatment against the possible hazards.

There is no definitive evidence that the concomitant administration of histamine H2-receptor antagonists and/or antacids will either prevent the occurrence of gastrointestinal side effects or allow the continuation of therapy with ketorolac tromethamine when and if these adverse reactions appear.

Genitourinary

Some NSAIDs are associated with persistent urinary symptoms (bladder pain, dysuria, urinary frequency), haematuria or cystitis. The onset of these symptoms may occur at any time after the initiation of therapy with an NSAID. Some cases have become severe on continued treatment. Should urinary symptoms occur, in the absence of an alternate explanation, treatment with ketorolac tromethamine **must be stopped immediately** to obtain recovery. This should be done before urological investigations or treatments are carried out.

Haematologic

NSAIDs inhibiting prostaglandin biosynthesis interfere with platelet function to varying degrees; patients who may be adversely affected by such an action, such as those on anti-coagulants or suffering from haemophilia or platelet disorders should be carefully observed when ketorolac tromethamine is administered.

Ketorolac tromethamine inhibits platelet function and may prolong bleeding time (see

WARNINGS AND PRECAUTIONS: Anti-platelet Effects). It does not affect platelet count, prothrombin time (PT) or partial thromboplastin time (PTT).

Anti-coagulants: Numerous studies have shown that the concomitant use of NSAIDs and anticoagulants increases the risk of bleeding. Concurrent therapy of ketorolac tromethamine with warfarin requires close monitoring of the international normalized ratio (INR).

Even with therapeutic INR monitoring, increased bleeding may occur.

Use of ketorolac tromethamine in patients who are receiving therapy that affects haemostasis should be undertaken with caution, including close monitoring. The concurrent use of ketorolac tromethamine and prophylactic, low dose heparin (2500-5000 units q12h), warfarin and dextrans may also be associated with an increased risk of bleeding.

In patients receiving anticoagulants, the risk of intramuscular haematoma formation from Ketorolac Tromethamine injections is increased.

Prothrombin time should be carefully monitored in all patients receiving oral anticoagulant therapy concomitantly with ketorolac tromethamine.

Ketorolac Tromethamine Injection given with 2 doses of 5000 U of heparin to 11 healthy volunteers, resulted in a mean template bleeding time of 6.4 minutes (3.2-11.4 minutes) compared to a mean of 6.0 min (3.4-7.5 minutes) for heparin alone and 5.1 minutes (3.5-8.5 minutes) for placebo.

The *in vitro* binding of warfarin to plasma proteins is only slightly reduced by ketorolac tromethamine (99.5% control vs. 99.3%) at plasma concentrations of 5-10 μg/mL

Anti-platelet Effects: NSAIDs inhibit platelet aggregation and have been shown to prolong bleeding time in some patients. Unlike acetylsalicylic acid (ASA), their effect on platelet function is quantitatively less, or of shorter duration, and is reversible. The inhibition of platelet function by ketorolac tromethamine is normalized within 24 to 48 hours after the drug is discontinued.

Ketorolac tromethamine and other NSAIDs have no proven efficacy as anti-platelet agents and should NOT be used as a substitute for ASA or other anti-platelet agents for prophylaxis of cardiovascular thromboembolic diseases. Anti-platelet therapies (e.g. ASA) should NOT be discontinued. There is some evidence that use of NSAIDs with ASA can markedly attenuate the cardioprotective effects of ASA (see DRUG INTERACTIONS: Drug-Drug Interactions, Acetylsalicylic Acid (ASA) or other NSAIDs).

Concomitant administration of ketorolac tromethamine with low dose ASA increases the risk of GI ulceration and associated complications.

Blood dyscrasias: Blood dyscrasias (such as neutropenia, leukopenia, thrombocytopenia, aplastic anemia and agranulocytosis) associated with the use of NSAIDs are rare, but could occur with severe consequences.

Anemia is sometimes seen in patients receiving NSAIDs, including ketorolac tromethamine. This may be due to fluid retention, GI blood loss, or an incompletely described effect upon erythropoiesis. Patients on long-term treatment with NSAIDs, including ketorolac tromethamine should have their haemoglobin or haematocrit checked if they exhibit any signs or symptoms of anemia or blood loss.

Haemorrhage and perioperative use of Ketorolac Tromethamine Injection: Post-operative haematomas and other symptoms of wound bleeding have been reported in association with the perioperative use of intramuscular Ketorolac Tromethamine Injection. Physicians should be aware of the potential risk of bleeding when haemostasis is critical in cases such as, but not limited to, resection of the prostate, tonsillectomy or cosmetic surgery. Ketorolac tromethamine is contraindicated in patients who have coagulation disorders. If ketorolac tromethamine is to be administered to patients who are receiving drug therapy that interferes with haemostasis, careful observation is advised.

Hepatic / Biliary / Pancreatic

As with other NSAIDs, borderline elevations of one or more liver enzyme tests (AST, ALT, alkaline phosphatase) may occur in up to 15% of patients. These abnormalities may progress, may remain essentially unchanged, or may be transient with continued therapy.

Meaningful elevations (greater than 3 times normal) of serum transaminases (glutamate pyruvate [SGPT or ALT] and glutamic oxaloacetic [SGOT or AST]), occurred in clinical trials in less than 1% of patients.

A patient with symptoms and/or signs suggesting liver dysfunction, or in whom an abnormal liver function test has occurred, should be evaluated for evidence of the development of a more severe hepatic reaction while on therapy with this drug. Severe hepatic reactions including jaundice and cases of fatal hepatitis, liver necrosis and hepatic failure, some of them with fatal outcomes, have been reported with NSAIDs.

Although such reactions are rare, if abnormal liver tests persist or worsen, if clinical signs and symptoms consistent with liver disease develop (e.g. jaundice), or if systemic manifestations occur (e.g. eosinophilia, associated with rash, etc.), ketorolac tromethamine should be discontinued.

Ketorolac tromethamine is contraindicated in patients with severe liver impairment or active liver disease. If there is a need to prescribe this drug in the presence of impaired liver function, it must be done under strict observation. Caution should be observed if ketorolac tromethamine is to be used in patients with a history of liver disease. Patients with impaired hepatic function from cirrhosis do not have any clinically important changes in ketorolac tromethamine clearance (see ACTION AND CLINICAL PHARMACOLOGY: Special Populations and Conditions).

Studies in patients with active hepatitis or cholestasis have not been performed.

Hypersensitivity Reactions

Anaphylactoid Reactions: As with NSAIDs in general, anaphylactoid reactions have occurred in

patients without known prior exposure to ketorolac tromethamine. Counteractive measures must be available when administering the first dose of Ketorolac Tromethamine Injection. In post-marketing experience, rare cases of anaphylactic/ anaphylactoid reactions and angioedema have been reported in patients receiving ketorolac tromethamine. Ketorolac tromethamine should NOT be given to patients with the ASA-triad. This symptom complex typically occurs in asthmatic patients who experience rhinitis with or without nasal polyps, or who exhibit severe, potentially fatal bronchospasm after taking ASA or other NSAIDs (see CONTRAINDICATIONS).

ASA-Intolerance: Ketorolac tromethamine should NOT be given to patients with complete or partial syndrome of ASA-intolerance (rhinosinusitis, urticaria/angioedema, nasal polyps, asthma) in whom asthma, anaphylaxis, urticaria/angioedema, rhinitis or other allergic manifestations are precipitated by ASA or other NSAIDs. Fatal anaphylactoid reactions have occurred in such individuals. As well, individuals with the above medical problems are at risk of a severe reaction even if they have taken NSAIDs in the past without any adverse reaction (see CONTRAINDICATIONS).

Cross-sensitivity: Patients sensitive to one NSAID may be sensitive to any of the other NSAIDs as well.

Serious skin reactions: (See WARNINGS AND PRECAUTIONS: Skin)

Immune

(See WARNINGS AND PRECAUTIONS: Infection, Aseptic Meningitis)

Infection

Ketorolac tromethamine, in common with other NSAIDs, may mask signs and symptoms of an underlying infectious disease.

Aseptic Meningitis: Rarely, with some NSAIDs, the symptoms of aseptic meningitis (stiff neck, severe headaches, nausea and vomiting, fever or clouding of consciousness) have been observed. Patients with autoimmune disorders (systemic lupus erythematosus, mixed connective tissue diseases, etc.) seem to be pre-disposed. Therefore, in such patients, the health care provider must be vigilant to the development of this complication.

Neurologic

Some patients may experience drowsiness, dizziness, blurred vision, vertigo, tinnitus, hearing loss, insomnia or depression with the use of NSAIDs, such as ketorolac tromethamine. If patients experience such adverse reaction(s), they should exercise caution in carrying out activities that require alertness.

Ophthalmologic

Blurred and/or diminished vision has been reported with the use of NSAIDs. If such symptoms develop, ketorolac tromethamine should be discontinued and an ophthalmologic examination performed. Ophthalmologic examination should be carried out at periodic intervals in any patient receiving ketorolac tromethamine for an extended period of time.

Peri-Operative Considerations

(See CONTRAINDICATIONS: Coronary Artery Bypass Graft Surgery, see WARNINGS AND PRECAUTIONS: Haemorrhage and perioperative use of Ketorolac Tromethamine Injection)

Psychiatric
(See WARNINGS AND PRECAUTIONS: Neurologic)

Renal
Long term administration of NSAIDs to animals has resulted in renal papillary necrosis and other abnormal renal pathology. In humans, there have been reports of acute renal failure, acute interstitial nephritis, renal papillary necrosis, haematuria, low grade proteinuria and occasionally nephrotic syndrome.

Renal insufficiency due to NSAID use is seen in patients with pre-renal conditions leading to reduction in renal blood flow or blood volume. Under these circumstances, renal prostaglandins help maintain renal perfusion and glomerular filtration rate (GFR). In these patients, administration of a NSAID may cause a reduction in prostaglandin synthesis leading to impaired renal function. Patients at greatest risk of this reaction are those with pre-existing renal insufficiency (GFR < 60 mL/min or 1 mL/s), dehydrated patients, patients on salt restricted diets, those with congestive heart failure, cirrhosis, liver dysfunction, taking angiotensin-converting enzyme inhibitors, angiotensin-II receptor blockers, cyclosporin, diuretics, sepsis and those who are elderly. Serious or life-threatening renal failure has been reported in patients with normal or impaired renal function after short term therapy with NSAIDs. Even patients at risk who demonstrate the ability to tolerate a NSAID under stable conditions may decompensate during periods of added stress (e.g. dehydration due to gastroenteritis). Discontinuation of NSAIDs is usually followed by recovery to the pre-treatment state.

Caution should be used when initiating treatment with NSAIDs, such as ketorolac tromethamine, in patients with considerable dehydration. Such patients should be rehydrated prior to initiation of therapy. Caution is also recommended in patients with pre-existing kidney disease.

Elevations of blood urea nitrogen (BUN) and creatinine have been reported in clinical trials with ketorolac. Ketorolac tromethamine is CONTRAINDICATED in patients with moderate to severe renal impairment.

Advanced Renal Disease: (See CONTRAINDICATIONS)

Fluid and Electrolyte Balance: Use of NSAIDs, such as ketorolac tromethamine, can promote sodium retention in a dose-dependent manner, which can lead to fluid retention and edema, and consequences of increased blood pressure, edema, and exacerbation of congestive heart failure. NaCl retention, oliguria, elevations of serum urea nitrogen and creatinine have also been observed in patients treated with ketorolac tromethamine. Thus, caution should be exercised in prescribing ketorolac tromethamine in patients with a history of congestive heart failure, compromised cardiac function, cardiac decompensation, hypertension, increased age or other conditions predisposing to fluid retention (see WARNINGS AND PRECAUTIONS: Cardiovascular).

Use of NSAIDs, such as ketorolac tromethamine, can increase the risk of hyperkalemia, especially in patients with diabetes mellitus, renal failure, increased age, or those receiving concomitant therapy with adrenergic blockers, angiotensin-converting enzyme inhibitors, angiotensin-II receptor antagonists, cyclosporin, or some diuretics.

Electrolytes should be monitored periodically (see CONTRAINDICATIONS).

Respiratory
ASA-induced asthma is an uncommon but very important indication of ASA and NSAID sensitivity. It occurs more frequently in patients with asthma who have nasal polyps.

Sexual Function/Reproduction/Fertility
The use of ketorolac tromethamine, as with any drug known to inhibit cyclooxygenase/prostaglandin synthesis, may impair fertility and is not recommended in women attempting to conceive. Therefore, in women who have difficulties conceiving, or who are undergoing investigation of infertility, withdrawal of ketorolac tromethamine should be considered.

Skin
In rare cases, serious skin reactions, some of them fatal, such as Stevens-Johnson syndrome, toxic epidermal necrolysis, exfoliative dermatitis and erythema multiforme have been associated with the use of some NSAIDs. Because the rate of these reactions is low, they have usually been noted during post-marketing surveillance in patients taking other medications also associated with the potential development of these serious skin reactions. Thus, causality is NOT clear. These reactions are potentially life threatening but may be reversible if the causative agent is discontinued and appropriate treatment instituted. Patients should be advised that if they experience a skin rash they should discontinue their NSAID and contact their physician for assessment and advice, including which additional therapies to discontinue.

Special Populations

***Pregnant Women*: Ketorolac tromethamine is CONTRAINDICATED for use during the third trimester of pregnancy because of risk of premature closure of the ductus arteriosus and the potential to prolong parturition (see CONTRAINDICATIONS and TOXICOLOGY).**

Caution should be exercised in prescribing ketorolac tromethamine during the first and second trimesters of pregnancy (see TOXICOLOGY).

Inhibition of prostaglandin synthesis may adversely affect pregnancy and/or the embryo-foetal development. Data from epidemiological studies suggest an increased risk of miscarriage and of cardiac malformation after use of a prostaglandin synthesis inhibitor in early pregnancy.

In animals, administration of a prostaglandin synthesis inhibitor has been shown to result in increased pre- and post-implantation loss and embryo-foetal lethality. In addition, increased incidences of various malformations, including cardiovascular, have been reported in animals given a prostaglandin synthesis inhibitor during the organogenetic period.

Ketorolac tromethamine is not recommended in labour and delivery because, through their prostaglandin synthesis inhibitory effect, they may adversely affect fetal circulation and inhibit uterine contractions, thus increasing the risk of uterine hemorrhage (see CONTRAINDICATIONS).

Nursing Women: (see CONTRAINDICATIONS)

Pediatrics: (see CONTRAINDICATIONS)

Geriatrics: Patients older than 65 years (referred to in this document as older or elderly) and frail or debilitated patients are more susceptible to a variety of adverse reactions from NSAIDs. The incidence of these adverse reactions increases with dose and duration of treatment. In addition, these patients are less tolerant to ulceration and bleeding. Most reports of fatal GI events are in this population. Older patients are also at risk of lower esophageal injury including ulceration and bleeding. For such patients, consideration should be given to a starting dose lower than the one usually recommended, with individual adjustment when necessary and under close supervision.

Post-marketing experience with ketorolac tromethamine suggests that there may be a greater risk of gastrointestinal ulcerations, bleeding, and perforation in the elderly and most spontaneous reports of fatal gastrointestinal events are in this population. This is particularly true in elderly patients who receive an average daily dose greater than 60 mg/day of ketorolac tromethamine. Because ketorolac is cleared somewhat more slowly by the elderly (see PHARMACOKINETICS), extra caution and the lowest effective dose should be used (see DOSAGE AND ADMINISTRATION).

Monitoring and Laboratory Tests

The following testing or monitoring is recommended for various populations of patients taking ketorolac tromethamine. This is not an exhaustive list.

- Renal function parameters such as serum creatinine and serum urea (in case of co-prescription of anti-hypertensives, methotrexate, cyclosporine, adrenergic blockers and in susceptible patients regarding the renal effects of NSAIDS e.g. impaired renal function or dehydration). See CONTRAINDICATIONS, WARNINGS AND PRECAUTIONS: Renal, and DRUG INTERACTIONS.

- Blood pressure (in case of anti-hypertensives co-prescription, and in susceptible patients with fluid retention)

- INR/effects of anticoagulants (Co-prescription of oral anticoagulants). See WARNINGS AND PRECAUTIONS: Hematologic.

- Lithium plasma concentrations (in case of lithium co-prescription)

ADVERSE REACTIONS

Adverse Drug Reaction Overview

The most common adverse reactions encountered with non-steroidal anti-inflammatory drugs are gastrointestinal, of which peptic ulcer, with or without bleeding is the most severe. Fatalities have occurred, particularly in the elderly.

Clinical Trial Adverse Drug Reactions

Because clinical trials are conducted under very specific conditions the adverse reaction rates observed in the clinical trials may not reflect the rates observed in practice and should not be compared to the rates in the clinical trials of another drug. Adverse drug reaction information from clinical trials is useful for identifying drug-related adverse events and for approximating rates.

Ketorolac Tromethamine Injection

The adverse reactions listed below were reported in Ketorolac Tromethamine Injection clinical efficacy trials. In these trials patients (N=660) received either single 30 mg doses (N=151) or multiple 30 mg doses (N=509) over a time period of 5 days or less for pain resulting from surgery. These reactions may or may not be drug related.

Table 1: Most Common Clinical Trial Adverse Drug Reactions (10-13%, 4-9% and 2-3%)

Body System	Incidence	Adverse Reaction
**Nervous System**	10-13%	Somnolence
	4-9%	Headache
	2-3%	Sweating, dizziness
**Digestive System**	10-13%	Nausea
	4-9%	Vomiting
**Injection Site**	4-9%	Injection site pain
**Cardiovascular System**	2-3%	Vasodilation

Table 2: Less Common Clinical Trial Adverse Drug Reactions (< 1%)

**Nervous system**	insomnia, increased dry mouth, abnormal dreams, anxiety, depression, paraesthesia, nervousness, paranoid reaction, speech disorder, euphoria, libido increased, excessive thirst, inability to concentrate, stimulation
**Digestive system**	flatulence, anorexia, constipation, diarrhea, dyspepsia, gastrointestinal fullness, gastrointestinal haemorrhage, gastrointestinal pain, melena, sore throat, liver function abnormalities, rectal bleeding, stomatitis

**Cardiovascular system:**	hypertension, chest pain, tachycardia, haemorrhage, palpitation, pulmonary embolus, syncope, ventricular tachycardia, pallor, flushing
**Injection site**	injection site reaction
**Body as a Whole**	asthenia, fever, back pain, chills, pain, neck pain
**Special senses**	taste perversion, tinnitus, blurred vision, diplopia, retinal haemorrhage
**Musculo-skeletal system**	myalgia, twitching
**Respiratory system**	asthma, cough increased, dyspnea, epistaxis, hiccup, rhinitis
**Skin and appendages**	pruritus, rash, subcutaneous haematoma, skin disorder
**Urogenital system**	dysuria, urinary retention, oliguria, increased urinary frequency, vaginitis
**Metabolic/nutritional disorders**	edema, hypokalemia, hypovolemia
**Haematologic and lymphatic system**	anemia, coagulation disorder, purpura

Abnormal Haematologic and Clinical Chemistry Findings

Elevations of blood urea nitrogen (BUN) and creatinine have been reported in clinical trials with ketorolac.

Post-Market Adverse Drug Reactions

Additional reports of adverse events temporally associated with ketorolac tromethamine during worldwide post-marketing experience are included below. Because these events are reported voluntarily from a population of uncertain size, it is not always possible to reliably estimate their frequency or clearly establish a causal relationship to ketorolac tromethamine exposure.

The following post-marketing adverse experiences have been reported for patients who have received either formulation of ketorolac tromethamine:

Renal Events: acute renal failure, flank pain with or without haematuria and/or azotemia, nephritis, hyponatremia, hyperkalemia, hemolytic uremic syndrome, urinary retention.

Hypersensitivity Reactions: bronchospasm, laryngeal edema, asthma, hypotension, flushing, rash, anaphylaxis, angioedema and anaphylactoid reactions. Such reactions have occurred in patients with no prior history of hypersensitivity.

Gastrointestinal Events: gastrointestinal hemorrhage, peptic ulceration, gastrointestinal perforation, pancreatitis, melena, esophagitis, hematemesis.

Hematologic Events: postoperative wound haemorrhage, rarely requiring blood transfusion (see PRECAUTIONS), thrombocytopenia, epistaxis, leukopenia, hematomata, increased bleeding time.

Central Nervous System: Convulsions, abnormal dreams, hallucinations, hyperkinesia, hearing

loss, aseptic meningitis, extrapyramidal symptoms, psychotic reactions.

Hepatic Events: hepatitis, liver failure, cholestatic jaundice.

Cardiovascular: pulmonary edema, hypotension, flushing, bradycardia.

Reproductive, female: infertility.

Dermatology: Lyell's syndrome, Stevens-Johnson syndrome, exfoliative dermatitis, maculopapular rash, urticaria.

Body as Whole: infection.

Urogenital: interstitial nephritis, nephrotic syndrome, raised serum urea and creatinine.

DRUG INTERACTIONS

Drug-Drug Interactions

Acetylsalicylic acid (ASA) or other NSAIDs: The use of ketorolac tromethamine in addition to most NSAIDs, including over-the-counter ones (such as ibuprofen) for analgesic and/or anti-inflammatory effects is usually contraindicated because of the absence of any evidence demonstrating synergistic benefits and the potential for additive adverse reactions.

The exception is the use of low dose ASA for cardiovascular protection, when another NSAID is being used for its analgesic/anti-inflammatory effect, keeping in mind that combination NSAID therapy is associated with additive adverse reactions.

Some NSAIDs (e.g. ibuprofen) may interfere with the anti-platelet effects of low dose ASA, possibly by competing with ASA for access to the active site of cyclooxygenase-1.
In vitro studies indicated that, at therapeutic concentrations of salicylates (300 µg/mL), the binding of ketorolac tromethamine was reduced from approximately 99.2% to 97.5% representing a potential two-fold increase in unbound ketorolac tromethamine plasma levels.

Antacids: There is no definitive evidence that the concomitant administration of histamine H2-receptor antagonists and/or antacids will either prevent the occurrence of gastrointestinal side effects or allow the continuation of ketorolac tromethamine therapy when and if these adverse reactions appear.

Anti-coagulants: (See WARNINGS AND PRECAUTIONS: Haematologic, Anticoagulants).

Anti-hypertensives: NSAIDs may diminish the anti-hypertensive effects of angiotensin converting enzyme (ACE) inhibitors.

Combinations of ACE inhibitors, angiotensin-II antagonists, or diuretics with NSAIDs might have an increased risk for acute renal failure and hyperkalemia. Blood pressure and renal function (including electrolytes) should be monitored more closely in this situation, as occasionally there can be a substantial increase in blood pressure.

Anti-platelet Agents (including ASA): There is an increased risk of bleeding, via inhibition of

platelet function, when anti-platelet agents are combined with NSAIDs, such as ketorolac tromethamine (see WARNINGS AND PRECAUTIONS: Haematologic, Anti-platelet Effects).

Cyclosporin: (See WARNINGS AND PRECAUTIONS: Renal).

Digoxin: Concomitant administration of an NSAID with digoxin can result in an increase in digoxin concentrations which may result in digitalis toxicity. Increased monitoring and dosage adjustments of digitalis glycosides may be necessary during and following concurrent NSAID therapy. Ketorolac tromethamine does not alter digoxin protein binding.

Diuretics: Clinical studies as well as post-marketing observations have shown that NSAIDs can reduce the effect of diuretics.

Ketorolac tromethamine reduces the diuretic response to furosemide by approximately 20% in normovolemic subjects, so particular care should be taken in patients with cardiac decompensation.

Glucocorticoids: Some studies have shown that the concomitant use of NSAIDs and oral glucocorticoids increases the risk of GI adverse events such as ulceration and bleeding. This is especially the case in older (> 65 years of age) individuals.

Lithium: Monitoring of plasma lithium concentrations is advised when stopping or starting a NSAID, as increased lithium concentrations can occur. Some NSAIDs have been reported to inhibit renal lithium clearance, leading to an increase in plasma lithium concentrations and potential lithium toxicity. The effect of ketorolac tromethamine on lithium plasma levels has not been studied. Cases of increased lithium plasma concentrations during therapy with ketorolac tromethamine have been reported.

Methotrexate: Caution is advised in the concomitant administration of methotrexate and NSAIDs, as this has been reported to reduce the clearance of methotrexate, thus enhancing its toxicity. In case combination treatment with methotrexate and NSAIDs is necessary, blood cell count and the renal function should be monitored. Concomitant administration of NSAIDs with a potentially myelotoxic drug, such as methotrexate, appears to be a predisposing factor to the onset of a cytopenia.

Oxpentifylline: When Ketorolac Tromethamine Injection is administered concurrently with oxpentifylline, there is an increased tendency to bleeding. The concomitant use of ketorolac tromethamine and oxpentifylline is contraindicated.

Probenacid: concomitant administration of ketorolac tromethamine and probenacid results in the decreased clearance and volume of distribution of ketorolac and a significant increase in ketorolac plasma levels (approximately three-fold increase) and terminal half-life (approximately two-fold increase). The concomitant use of ketorolac tromethamine and probenacid is contraindicated.

Selective Serotonin Reuptake Inhibitors (SSRIs): Concomitant administration of NSAIDs and SSRIs may increase the risk of gastrointestinal ulceration and bleeding (see WARNINGS AND PRECAUTIONS: Gastrointestinal).

Drug-Food Interactions
Interactions with food have not been established.

Drug-Herb Interactions
Interactions with herbal products have not been established.

Drug-Laboratory Interactions
Interactions with laboratory tests have not been established.

Drug-Lifestyle Interactions
Potential Effects on Driving and Using Machinery: Some patients may experience drowsiness dizziness vertigo insomnia or depression with the use of ketorolac tromethamine. Therefore, patients should exercise caution in carrying out potentially hazardous activities that require alertness.

DOSAGE AND ADMINISTRATION

Dosing Considerations
Use of ketorolac tromethamine should be limited to the lowest effective dose for the shortest possible duration of treatment (see INDICATIONS AND CLINICAL USE).

In no case is the duration of ketorolac tromethamine treatment to exceed 7 days.

Recommended Dose and Dosage Adjustment
Adults (>18 years of age): Dosage should be adjusted according to the severity of the pain and the response of the patient.

Intramuscular: The recommended usual initial dose is 10-30 mg, according to pain severity. Subsequent dosing may be 10 mg to 30 mg every 4-6 hours as needed to control pain. The lowest effective dose should be administered.

The administration of Ketorolac Tromethamine Injection should be limited to short-term therapy (not over 2 days). The total daily dose should not exceed 120 mg because the risk of toxicity appears to increase with longer use at recommended doses (see WARNINGS and PRECAUTIONS). The administration of continuous multiple daily doses of Ketorolac Tromethamine Injection has not been extensively studied. There has been limited experience with intramuscular dosing for more than 3 days since the vast majority of patients have transferred to oral medication or no longer required analgesic therapy after this time.

Conversion from Parenteral to Oral Therapy
When ketorolac tromethamine tablets are used as a follow-on therapy to parenteral ketorolac, the total combined daily dose of ketorolac (oral + parenteral) should not exceed 120 mg in younger adult patients or 60 mg in elderly patients on the day the change of formulation is made. On subsequent days, oral dosing should not exceed the recommended daily maximum of 40 mg. Ketorolac Tromethamine Injection should be replaced by an oral analgesic as soon as feasible.

The total duration of combined intramuscular and oral treatment should not exceed 5 days.

Renal Impairment
Ketorolac tromethamine is contraindicated in patients with moderate to severe renal impairment (serum creatinine >442 μmol/L). Ketorolac tromethamine should be used with caution in patients with lesser renal impairment (serum creatinine 170 - 442 μmol/L). Such patients should receive a reduced dose of ketorolac tromethamine, and their renal status should be closely monitored. It is recommended that the daily dose be reduced by half; a total daily dose of 60 mg should not be exceeded. Dialysis does not significantly clear ketorolac from bloodstream.

See CONTRAINDICATIONS, WARNINGS AND PRECAUTIONS: Renal and ACTION AND CLINICAL PHARMACOLOGY: Special Populations and Conditions, Renal Insufficiency.

Hepatic Impairment
Ketorolac tromethamine is contraindicated in patients with severe liver impairment or active liver disease. Caution should be observed in giving ketorolac tromethamine to patient with mild to moderate hepatic insufficiency.

See CONTRAINDICATIONS, WARNINGS AND PRECAUTIONS: Hepatic/Biliary/Pancreatic and ACTION AND CLINICAL PHARMACOLOGY: Special Populations and Conditions, Hepatic Insufficiency.

Elderly, Frail or Debilitated Patients
These patients are at increased risk of the serious consequences of adverse reactions.

Parenteral: The lower end of the dosage range is recommended. The initial dose should be 10 mg. The total daily dose of Ketorolac Tromethamine Injection in the elderly should not exceed 60 mg.

<u>Missed Dose</u>
The missed dose should be administered as soon as remembered, and then the regular dosing schedule should be continued. Two doses of ketorolac tromethamine should not be administered at the same time.

<u>Administration</u>
Parenteral drug products should be inspected visually for particulate material and discoloration prior to use.

OVERDOSAGE

Signs and Symptoms: Overdoses of ketorolac tromethamine have been variously associated with abdominal pain, nausea, vomiting, hyperventilation, peptic ulcers and/or erosive gastritis, gastrointestinal bleeding, and renal dysfunction which have generally resolved after discontinuation of dosing. Metabolic acidosis has been reported following intentional overdosage. Although rare, hypertension, acute renal failure, respiratory depression, coma and death have been reported after significant overdose of NSAIDs. Anaphylactoid reactions have

been reported with therapeutic ingestion of NSAIDs and may occur following an overdose.

In a gastroscopic study of healthy subjects, daily doses of 360 mg given over an 8-hour interval for each of five consecutive days (3 times the highest recommended dose) caused pain and peptic ulcers which resolved after discontinuation of dosing

Treatment
Patients should be managed by symptomatic and supportive care following overdose. There are no specific antidotes. Dialysis does not significantly clear ketorolac from the bloodstream.

For management of a suspected drug overdose contact your regional Poison Control Centre.

ACTION AND CLINICAL PHARMACOLOGY

Pain relief is comparable following the administration of ketorolac by intramuscular or oral routes. The peak analgesic effect occurs at 2-3 hours post-dosing with no evidence of a statistically significant difference over the recommended dosage range. The greatest difference between large and small doses of ketorolac tromethamine administered by either route is in the duration of analgesia.

Mechanism of Action

Ketorolac tromethamine s a non-steroidal anti-inflammatory drug (NSAID) that exhibits analgesic activity mediated by peripheral effects. The mechanism of action of ketorolac, like that of other NSAIDS, is not completely understood, but is believed to be related to prostaglandin synthetase inhibition.

Pharmacodynamics

(See DETAILED PHARMACOLOGY)

Pharmacokinetics

The pharmacokinetics are linear following single and multiple dosing. Steady state plasma levels are attained after one day of Q.I.D. dosing.

Following intramuscular administration, peak plasma concentrations of 2.2 to 3.0 μg/mL occur an average of 50 minutes after a single 30 mg dose. The terminal plasma half-life ranges between 3.5 and 9.2 hours in young adults and between 4.7 and 8.6 hours in elderly subjects (mean age = 72 years).

In renally impaired patients there is a reduction in clearance and an increase in the terminal half-life of ketorolac tromethamine (see table 3 below).

The parenteral administration of ketorolac tromethamine has not been demonstrated to affect the haemodynamics of anaesthetized patients.

A series of studies were carried out in mice, rats, rabbits, monkeys and humans to characterize the pharmacokinetic profile of the free acid of ketorolac and ketorolac tromethamine. The salt

form of the compound was later selected for development due to its more rapid and complete absorption.

Absorption: Ketorolac tromethamine was rapidly (Tmax ranged from 0.25 to 1.5 hours) and completely absorbed after oral and IM doses in animals (>87%) and humans (>99%).

Distribution: The volume of distribution of ketorolac was estimated following intravenous dosing and ranged from 0.09 L/kg in mice to 0.38 L/kg in rats; in humans it averaged 0.15 L/kg.

Ketorolac was highly protein bound in human (99.2%), monkey (98.3%) and rabbit (98.2%) plasma; moderately bound in rat plasma (92.1%); and poorly bound in mouse plasma (72.0%). Binding was concentration independent in all species studied.

The tissue distribution of ketorolac-associated radioactivity was studied in male mice. The highest levels were found in the kidney which was the only organ which exceeded plasma levels at all time points (by about 50%). The lowest levels were present in the brain. However, all tissues eliminated ketorolac-associated radioactivity rapidly with a tissue half-life of <3.6 hours.

Distribution studies in pregnant rabbits and rats showed that ketorolac-associated radioactivity distributed into the fetus in low but measurable levels – less than 15% in rabbits and 6% in rats based upon fetal to maternal plasma or blood concentration ratios. Ketorolac-associated radioactivity was also passed into the milk of lactating animals. In rats, radioactivity concentrations in milk exceeded plasma concentrations at all time points by as much as four-fold. However, in rabbits, milk concentrations were only about 12% of plasma concentrations.

Clearance and Half-life: The pharmacokinetics of ketorolac in man following single or multiple intramuscular doses are linear. Steady state plasma levels are achieved after dosing every 6 hours for one day. No changes in clearance occurred with chronic dosing. The plasma half-life of ketorolac ranged from 2.1 hours in rabbits to 6.6 hours in rhesus monkeys and 7.7 hours in mice. In humans, the plasma half-life averaged 6.0 hours. Total plasma clearance ranged from 0.44 mL/min/kg in mice to 2.44 mL/min/kg in rats and averaged 0.35 mL/min/kg in humans.

Metabolism: Ketorolac is largely metabolized in the liver. The major metabolic path of ketorolac in humans is glucuronic acid conjugation. P-hydroxylation is an additional minor pathway.

In vitro and *in vivo* studies demonstrated that ketorolac does not induce or inhibit its own metabolism or the metabolism other drugs such as aniline, ethylmorphine and hexobarbital, upon multiple dosing.

A moderate first pass metabolism (about 20%) was observed in humans, while rabbits exhibited more extensive first pass metabolism (about 50%) following oral doses.

The metabolism and excretion patterns of ketorolac and its metabolites were similar following p.o., i.v. and i.m. dosing in the species studied. Ketorolac accounted for most of the radioactivity circulating in the plasma ranging from 79% in rabbits to 99% in mice and averaged 96% in humans. Conjugates of ketorolac were not detected in plasma in appreciable amounts in any

species. However, the p-hydroxy metabolite (which is essentially inactive when compared to ketorolac) was detected in the plasma of rats, rabbits and humans. Ketorolac and its metabolites were excreted predominantly in the urine of all species, ranging from 69% in rats to essentially 100% in the cynomolgus monkey and averaged 92% in humans. The most comparable species with respect to man metabolically was the mouse.

Elimination/Excretion: The primary route of excretion of ketorolac tromethamine and its metabolites (conjugates and the p-hydroxy metabolite) is in the urine (91.4%) with the remainder (6.1%) being excreted in the feces.

Special Populations and Conditions

Geriatrics (>65 years of age): The terminal plasma half-life of ketorolac is prolonged compared to young healthy volunteers to an average of 7 hours (ranging from 4.3 to 8.6 hours). The total plasma clearance may be reduced compared to young healthy volunteers, on average to 0.019 L/h/kg.

Hepatic Insufficiency: Patients with impaired hepatic function do not have any clinically important changes in ketorolac pharmacokinetics, although there is a statistically significant prolongation of Tmax and terminal phase half-life compared to young healthy volunteers.

Renal Insufficiency: Elimination of ketorolac is decreased in patients with renal impairment as reflected by a prolonged plasma half-life and reduced total plasma clearance when compared to young healthy subjects. The rate of elimination is reduced roughly in proportion to the degree of renal impairment except for patients who are severely renally impaired, in whom there is higher plasma clearance of ketorolac than estimated from the degree of renal impairment alone.

Table 3: The influence of age, liver and kidney function on the clearance and terminal half-life of Ketorolac Tromethamine Injection[1]

Types of Subjects	Total Clearance (in L/h/kg)[2]	Terminal Half Life (in hours)
	MEAN (range)	MEAN (range)
**Normal Subjects** (n=54)	0.023 (0.010-0.046)	5.3 (3.5-9.2)
**Healthy Elderly Subjects** (n=13) (mean age = 72, range = 65- 78)	0.019 (0.013-0.034)	7.0 (4.7-8.6)
**Patients with Hepatic Dysfunction** (n=13)	0.029 (0.013-0.066)	5.4 (2.2-6.9)
**Patients with Renal Impairment** (n=9) (serum creatinine 1.9-5.0 mg/dL)	0.014 (0.007-0.043)	10.3 (8.1-15.7)
**Renal Dialysis Patients** IM (n=9)	0.016 (0.003-0.036)	13.6 (8.0-39.1)

[1] Estimated from 30 mg single IM doses of ketorolac tromethamine
[2] Litres/hour/kilogram

STORAGE AND STABILITY

Ketorolac Tromethamine Injection, USP

Store at room temperature (15-30 °C) with protection from light.

Keep out of reach of children.

SPECIAL HANDLING INSTRUCTIONS

Parenteral drug products should be inspected visually for particulate matter and discoloration prior to administration whenever solution and container permit. Solutions which are not clear, or contain visible particulates, should not be used.

DOSAGE FORMS, COMPOSITION AND PACKAGING

Ketorolac Tromethamine Injection USP 30 mg/mL, is available in single use 1 mL vials, box of 10

Ketorolac Tromethamine Injection, USP

Ketorolac Tromethamine Injection USP, 30 mg/mL, available for intramuscular administration, is a clear and slightly yellow sterile solution. Each mL contains: ketorolac tromethamine 30 mg, ethyl alcohol 10% (w/v), sodium chloride for tonicity, sodium hydroxide and/or hydrochloric acid to adjust pH and water for injection. The product is preservative free.

Latex-Free Stoppers – Stoppers contain no dry natural rubber.

PART II: SCIENTIFIC INFORMATION

PHARMACEUTICAL INFORMATION

Drug Substance

Proper name: ketorolac tromethamine

Chemical name: (±)-5-Benzoyl-2, 3-dihydro-1H-pyrrolizine-1-carboxylic acid, 2-amino-2-(hydroxymethyl)-1, 3-propanediol

Molecular formula and molecular mass: $C_{19}H_{24}N_{2}0_{6}$; 376.41

Structural formula:

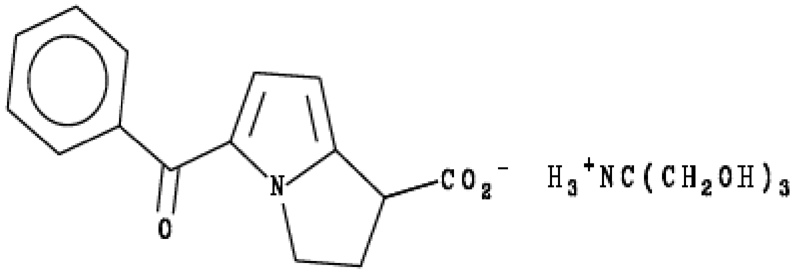

Physicochemical properties: ketorolac tromethamine (pKa = 3.46) is an off-white to white crystalline powder. It is freely soluble in water and methanol, slightly soluble in tetrahydrofuran, 190 proof and 200 proof ethanol and practically insoluble or insoluble in acetone, dichloromethane, toluene, ethylacetate, dioxane, hexane, butanol, and acetonitrile. The pH of a 1% (w/v) solution in distilled water is 5.7-6.7. Its melting point is about 162°C with decomposition.

DETAILED PHARMACOLOGY

Animal Pharmacology

Analgesic Properties:

Ketorolac is a potent orally active analgesic agent in tests utilizing an underlying inflammatory state. In mice, given oral or subcutaneous doses ranging from 0.05-2.25 mg/kg, the compound was 250-350 times more potent than ASA in inhibiting phenylquinone-induced writhing. Using a similar test in rats which received 0.03-1.0 mg/kg p.o., ketorolac was 180 times as potent as aspirin in inhibiting the writhing response.

In rats having adjuvant-induced arthritis, ketorolac p.o. was 400-800 times more potent than aspirin and twice as potent as naproxen in alleviating pain. The compound also significantly increased the pain threshold in yeast-inflamed paws of rats which were compressed at a constant rate of pressure (Randall-Selitto Test), its potency being 3 to 10 times that of naproxen.

The fact that ketorolac does not increase the pain threshold of the non-inflamed paw and does not exhibit analgesic activity in the mouse hot plate test indicates that it is not a morphine like compound.

Anti-inflammatory Properties:

Ketorolac displayed anti-inflammatory properties when tested in classical rat models to test intrinsic anti-inflammatory actions. The free acid form of the compound had approximately 36 times the anti-inflammatory potency of phenylbutazone, while the tromethamine salt was 118 times as active as phenylbutazone in inhibiting carrageenin-induced paw inflammation when administered orally. This difference in potency is due to the compound.

Ketorolac was weakly effective in inhibiting the development of ultraviolet-induced erythema when applied topically at a dose of 1 mg to guinea pigs. In the rat, however, topical application at dose levels of 0.01 and 0.1 mg/rat, was very effective in suppressing the heat induced local inflammatory reaction.

When administered to rats at a dose of 2 mg/kg/day p.o., for 6 days, ketorolac did not produce thymic involution. This indicates that the anti-inflammatory activity is not due to intrinsic corticosteroid activity in the molecule nor due to the stimulation of endogenous corticosteroid production. These findings were further confirmed by the dose-related anti-inflammatory activity in adrenalectomized rats.

Antipyretic Properties:

When administered orally to yeast-infected rats in doses ranging from 0.1-2.7 mg/kg, ketorolac had 20 times the antipyretic potency of aspirin.

Prostaglandin Inhibition:

There is substantial evidence in the literature to suggest that the anti-inflammatory, analgesic and antipyretic activities of non-steroidal anti-inflammatory drugs (NSAIDs) are due to their ability to inhibit prostaglandin biosynthesis.

Ketorolac, like other NSAIDs, inhibited the prostaglandin synthetase activity in bovine seminal vesicle microsomes, rabbit renal medullary microsomes, and human platelet microsomes, having substantially greater potency (1.0 to 5.3 times) than indomethacin.

Platelet Effects:

In *in vitro* studies, ketorolac was 37 times as active as aspirin in inhibiting aggregation of human platelets induced by collagen and 28 times more potent than aspirin in inhibiting arachidonic acid-induced platelet aggregation. However, ketorolac did not inhibit the primary phase of adenosine diphosphate-induced aggregation nor the aggregation elicited by thromboxane A2.

Central Nervous System Effects:

The acute intraperitoneal administration of ketorolac to mice had minimal behavioral effects at doses up to 300 mg/kg. Above this dose level, depression of normal behavior was seen.

No appreciable central nervous system (CNS) activity was produced by ketorolac. It did not possess anticonvulsant activity in mice in the maximal electroshock test nor did it inhibit pentylenetetrazol-induced seizures in mice or rats.

In mice, hexobarbital-induced sleep time was unaltered by ketorolac suggesting that the compound was not a CNS depressant.

The gross behavior and sleep patterns of cats dosed at up to 10 mg/kg, i.v., were unchanged.

Cardiovascular Effects:

Sequential administration of 1, 3 and 10 mg/kg, i.v. of ketorolac to anesthetized cats, produced minimal cardiovascular or autonomic responses.

In anesthetized dogs, doses of 1 to 30 mg/kg, i.v., produced inconsistent and variable changes in the cardiac contractile force, heart rate and blood pressure. The cardiovascular responses to adrenaline, nor-adrenaline, tyramine, phenylephrine and bilateral carotid artery occlusion were inhibited by ketorolac, suggesting that the compound may possess mild alpha-adrenoceptor blocking activity.

Bronchial Effects:

Ketorolac, when administered intravenously to guinea pigs in doses of 0.01-10 mg/kg failed to block histamine- or methacholine-induced bronchoconstriction. In the rat, the compound blocked methacholine-induced airway constriction (ED50 = 0.5 mg/kg).

Gastric Effects:

Doses of ketorolac at 0.1 and 1.0 mg/kg p.o. in rats did not alter significantly either the gastric juice volume or the total mEq of hydrogen ions secreted in response to histamine stimulation. Moreover, in common with other NSAIDs, both the acid and the tromethamine salt of ketorolac had a similar propensity to cause gastrointestinal erosions in rats independent of the route of administration

TOXICOLOGY

Acute Toxicity Studies

Animal	Strain	Sex	Route	LD 50 (mg/kg)
Mouse	HLA-SW/ICR	F	Oral	approx. 400
Mouse	HLA-SW/ICR	M/F	Oral+	529 (281-1540)*
Rat	COX-SD	F	Oral	112 (68-191)*
Rat	COX-SD	M/F	Oral+	100-400
Mouse	HLA-SW/ICR	F	i.p.	>400
Mouse	HLA-SW/ICR	M/F	i.p.+	473 (315-771)*
Rat	COX-SD	F	i.p.	158 (101-248)*
Rat	COX-SD	M/F	i.p.+	100-400

Note:* 95% confidence interval

+ studies with ketorolac tromethamine; all others with ketorolac free acid. All doses were administered in solution form.

Administration of the free acid of ketorolac at a dose of 200 mg/kg, p.o. in 1 male and 1 female cynomolgus monkey caused both monkeys to vomit after dosing. Other changes seen in the female included diarrhea and anorexia starting 5 days after dosing. The male monkey gained weight while the female had weight loss. Both animals had decreased haemoglobin and haematocrit and survived the 2 week post dose period.

In another study, the identical dose of ketorolac tromethamine salt caused vomiting in the female. No other clinical signs were recorded for this animal. The male monkey appeared normal throughout the study duration.

Sensitization:

The sensitization potential of a 0.1% solution of ketorolac tromethamine was evaluated in male guinea pigs. Ketorolac tromethamine did not cause sensitization when tested in the guinea pig model.

Vein Irritation:

An intravenous formulation containing ketorolac tromethamine at a concentration of 10 mg/mL was injected into the marginal ear vein of the left ear of each of 6 rabbits (New Zealand albino). The right ear served as a sham control. No evidence of vein irritation was seen following gross or microscopic pathological examinations.

An intravenous formulation containing 10% ethanol and ketorolac tromethamine at a concentration of 10 or 30 mg/mL was injected into the marginal ear vein of the left ears of 6 rabbits (New Zealand albino). The right ear received vehicle only. There was no evidence of

drug-related irritation in-life. Minimal irritation was noted microscopically in some animals that received the vehicle or drug formulations.

Subchronic Toxicity Studies

Oral:

Ketorolac was administered to groups of male and female mice at doses of 0 (vehicle control), 0.25, 1.0, 4.0 or 16.0 mg/kg/day for a period of 4 weeks.

No drug related change was seen in the mice receiving 0.25 mg/kg/day. In mice receiving the higher doses, dose related changes included decreased activity, pallor, unthrifty appearance, wasting and rough coat. Treatment related deaths occurred in the high dose (16 mg/kg/day) group only (4/6 males and 5/6 females). Food intakes of the female mice in groups receiving 1.0 or 4.0 mg/kg/day were significantly lower than control values. In treated male groups, food intakes were comparable to control values throughout the study.

Haematologic parameters measured revealed decreased haemoglobin and haematocrit levels for groups receiving 4.0 or 16.0 mg/kg/day and elevated total leukocyte and neutrophil counts in the high dose group animals. No biologically meaningful changes were found in any of the plasma chemistry parameters or urinalysis. Gastrointestinal inflammation, erosions and/or ulcers were present in the high dose animals only. No drug related pathological change was present in mice from other dose groups.

Daily oral administration of ketorolac to monkeys at doses of 0.0 (vehicle control), 0.5, 2, 8 or 32 mg/kg/day for 4 weeks resulted in clinical signs of toxicity and haematologic and pathologic effects at all dose levels. Clinically, a few isolated instances of dark colored urine, vomiting and dark colored feces (fecal blood) were seen in all dose groups but not in controls. There was a slight decrease in haemoglobin and haematocrit levels mainly in the high dose group animals. Other parameters, such as body weight, ophthalmoscopy, clinical chemistry and urinalysis were all comparable to control values. Gastric erosions were observed in some animals at all dose levels, while gastric ulceration and haemorrhage were seen in some animals receiving 8 or 32 mg/kg/day. Chronic colitis was seen in 3 out of 4 monkeys treated with the highest dose.

Intravenous:

Intravenous administration of ketorolac tromethamine to rabbits and monkeys at doses of 0 (vehicle), 0.5, 1.25 or 2.5 mg/kg/day for 2 weeks was well tolerated with no clinically significant treatment related effects.

Intramuscular:

Rabbits were administered ketorolac tromethamine intramuscularly at daily doses of 0 (saline control), 10 or 15 mg for 29 consecutive days. Each group comprising 3 males and 3 females received a dose volume of 0.5 mL/animal.

There were no treatment related clinical changes during the study. Minimal to slight haematologic changes occurred in some treated animals. Gross and/or microscopic examinations of the injection sites revealed focal haemorrhage, muscle fiber degeneration and mixed leukocyte infiltration in all groups.

Five groups, each comprised of 3 male and 3 female cynomolgus monkeys, were administered intramuscular injections of saline, vehicle or 4.5, 9.0 or 13.5 mg/kg/day of ketorolac tromethamine for 3 months. Injections were given thrice daily with dose volumes of 0.15, 0.15, 0.05, 0.10 or 0.15 mL/kg/dose for saline, vehicle, low, mid and high dose groups, respectively. The sites injected on the first day and last 7 days of injections were noted for histological examination.

There were no clinical signs of drug related systemic toxicity. However, the incidence and severity of lacerations and ulcers of the extremities (limbs and tail) were increased in the drug treated groups compared to the controls. These lesions were probably the result of bite wounds and the analgesic effect of the drug may have reduced the normal avoidance behavior in response to painful stimuli.

No drug related changes in body weight gain, eye morphology or clinical pathologic results were observed except for slight increases in blood urea nitrogen (BUN) in high and mid dose females.

Local irritation at the injection site was noted in animals from all treatment groups. In conclusion, doses of 4.5, 9.0, and 13.5 mg/kg of ketorolac tromethamine given to monkeys by three times daily intramuscular injections for 3 months caused essentially no drug related systemic toxicity.

Chronic Toxicity Studies

Mice (30 males and 30 females per group) were given either a placebo diet or drug-diet mixtures equivalent to an estimated daily dose of 0 (placebo), 3.3, 10 or 30 mg ketorolac tromethamine/kg/day for 6 months.

Treatment related clinical changes were seen in animals in the mid and high dose groups and these included pallor, rough coat, unthrifty appearance, wasting, abdominal enlargement, decreased activity, labored respiration and decreased body temperature. In general, trends of slightly lower body weight and lesser feed intake were observed in treated males and females relative to controls. No drug related ocular lesions were observed in animals.

Prior to termination of the study, 3 of 6 low dose, 9 of 60 mid dose and 52 of 60 high dose animals either died or had to be sacrificed because of poor clinical condition. The cause of debilitation or death of most of the mid and high dose animals was related to erosions and ulcerations in the stomach and large and/or small intestines. Many of these animals were anemic. At all dose levels, renal inflammatory lesions, especially in females were found. An apparent interruption of ovarian cyclic activity was noted histologically. Prostaglandin synthetase inhibitors have been reported to block ovulation by central activity.

Cynomolgus monkeys (4 males and 4 females/group) were administered ketorolac tromethamine orally, twice daily for a period of 6 months at doses of 0 (vehicle control), 0.75, 2.95 or 11.75 mg/kg/day.

There were no treatment related clinical changes or changes in laboratory tests with the exception of slightly elevated urea nitrogen levels in the ketorolac treated animals. The principal gross pathologic finding was pallor of the renal papilla and cortex in both males and females that received the test compound. The gross changes correlated microscopically with minimal to mild increases in interstitial matrix in the renal papilla of the mid and high dose animals only. No specific microscopic change was present in renal cortex which correlated with cortical pallor.

Two groups each with 5 male and 5 female cynomolgus monkeys were administered once daily 0.75 or 2.62 mg/kg of ketorolac tromethamine for 12 months. Two additional groups each with 8 males and 8 females received vehicle only or 9 mg/kg of ketorolac tromethamine for 12 months. All groups received 1.5 mL/kg/day of formulation administered into the stomach by nasal catheter. Three males and three female monkeys from the high dose and vehicle treated groups had a recovery period from dosing of months and then were given clinical laboratory analysis and a complete necropsy at the end of the 12 month dosing period.

Two females (one control and one mid-dose diagnosed with gastroenteropathy and enteropathy respectively) were sacrificed in a moribund condition at week 11 while one female diagnosed with pneumonia was sacrificed at study week 31. Causes of death were varied and not considered related to the test compound.

There were no drug related differences in the clinical condition of the surviving animals. The males showed a dose related decrease in RBC count, haemoglobin, haematocrit, mean corpuscular haemoglobin and haemoglobin concentration. The females were not affected to the same extent as the males but did show marginal decreases in some parameters at some time intervals (mainly in the highest dose group). Normalization of these tests occurred in animals after a 2 month drug free recovery period. The males had a significant increase in BUN, the magnitude of which increased with the dose and time of exposure to the drug. The females had no change in BUN, but the high dose group had a significant increase in serum creatinine at the 9 and 12 month intervals.

Oral administration of 9 mg/kg of ketorolac tromethamine for 12 months caused minimal renal microscopic pathologic changes which included increased intertubular matrix in the papilla and intratubular mineralization in the cortical, medullary and papillary tubules. Those animals given a 2 month period of recovery from dosing showed absences of morphologic damage.

These findings suggest that only mild, reversible kidney changes occurred with high doses of ketorolac tromethamine after one year of treatment. This conclusion is supported by the minimal histopathologic effects observed and by the absence of effects after the recovery period.

Carcinogenicity

The carcinogenic potential of ketorolac tromethamine was assessed in an 18 month feeding study. Fifty Swiss-Webster albino mice were randomly assigned to receive 0.5, 1.0 or 2.0 mg/kg/day of ketorolac tromethamine in their diet. A control group of 100 animals of each sex received the same diet without ketorolac. The duration of the study was 78 weeks. However, males in the highest dose group received control diet for the last 3 weeks of the study due to the high mortality rate in that group relative to controls. Female survival was not affected. All animals received a complete necropsy.

The average body weight of the high dose males was generally lower than that of the controls during the second half of the study. No such effect was evident in males in the lower dose groups or in females. Since the average food intake was similar for all dose groups throughout the study, the difference in body weight was not the result of reduced food intake.

Histopathologic examinations revealed no treatment related increase in the incidence of any type of tumor. Enteritis, gastroenteropathy and peritonitis were seen primarily in the high dose group and were considered expected sequelae to high doses of an NSAID.

In conclusion, there was no evidence for a carcinogenic effect of ketorolac tromethamine in the mouse.

A 24 month feeding study was conducted in rats to assess the carcinogenic potential of ketorolac tromethamine. Fifty Sprague-Dawley rats of either sex were administered in their diet either 0.8, 2.0 or 5.0 mg ketorolac/kg body weight. A control group of 100 animals received the same diet without the drug.

No treatment related changes were noted in clinical condition except for a reddish discoloration of the urine which occurred more frequently in treated males than in controls. The survival times were significantly lower than controls in high dose males and mid and high dose females.

The body weights of the high dose group females were approximately 10% lower than the controls during the last 6 months of the study although no differences in food intakes were noted among the various groups. The high dose males had decreased erythroid parameters, elevated platelet count and a higher incidence of blood in the urine specimens. High dose males and females had elevated BUN and increased neutrophil and decreased lymphocyte counts. Mid and high dose females had a lower urinary specific gravity compared to control females.

There was no evidence for a carcinogenic effect of ketorolac tromethamine in rats.

Mutagenicity

In vitro mutagenic studies were performed with ketorolac, ketorolac tromethamine and tromethamine using 5 strains of bacteria and one of yeast.

Tests were carried out with and without mammalian microsomal activation. None of the compounds tested were mutagenic in any of these test systems. Ketorolac tromethamine was also negative in the in vivo mouse micronucleus test.

Fertility and Reproduction

Female Rat:

A two generation study was conducted to evaluate the effects of ketorolac tromethamine on fertility and reproduction in female rats. Groups, each composed of 40 female rats, were administered drug-diet mixtures to achieve doses of 0 (placebo control), 1, 4 or 16 mg/kg/day. The P1 female rats were treated from 14 days before mating until gestation day 13 or until the F1 pups were weaned at 21 days postpartum. The reproductive performance of F2 pups was also evaluated.

No treatment-related effects were seen on the reproductive status at gestation day 13. Some treated females died during the study and the deaths were attributed to gastroenteropathy, nephropathy, or dystocia.

The length of gestation was significantly increased in the high-dose (P1 females) group (median 25 days) when compared to the controls (median 22 days). A slight increase in the length of gestation (median 22.5 days) was noted in the mid-dose group when compared to the controls. Decreased live litter sizes and survival indices were noted in the high-dose group when compared to controls. No pups from the high-dose group survived to day 4 of postnatal life. Decreased survival indices (up to day 7) were noted in the mid-dose group when compared to controls. The maternal care and lactation data were comparable among the control, low and mid- dose groups. The clinical condition and body weights of surviving F1 pups were comparable among all groups. The postnatal behavioral and developmental evaluation of F1 pups indicated no treatment-related effects. The reproductive performance of the F1 pups and the neonatal survival of their offspring (F2 pups) were comparable among the groups.

In conclusion, dietary administration of ketorolac tromethamine to female rats prior to and during mating, gestation, parturition and lactation resulted in increased mortality among F0 dams and reduced F1 litter size at 16 mg/kg/day and prolonged gestation period and reduced neonatal survival at 4 and 16 mg/kg/day.

Male Rat:

Four groups each with 25 male rats were dosed once daily by gavage with 0, 3.0, 6.0 or 9.0 mg/kg of ketorolac tromethamine. Males were dosed for 104 days prior to cohabitation with undosed females and continued to be dosed through the 14 day mating period. Mating units consisted of one dosed male and two untreated females. Approximately half of the females with evidence of mating were sacrificed at midgestation while the other half were allowed to litter and raise their pups until 21 days postpartum.

No drug-related changes in the clinical condition of the males were observed. Body weight and food intake were not affected by drug treatment. There were no drug-related differences in the number of males leaving evidence of mating, the pre-coital interval, or in the number impregnating females.

The females mated with high-dose males and sacrificed at midgestation had a significant preimplantation loss resulting in smaller litter sizes. However, there was no increase in the number of resorptions (post implantation loss) and no decreases in litter size of dams littering at term. Therefore, the reduced number of implantations in the high dose females was not considered to be a drug effect.

There were no differences between drug groups and the control group in regard to body weight, length of gestation, gestation index, lactation index, number of pups born alive and survival indices. Thus, administration of ketorolac tromethamine by gavage to male rats prior to and during the mating period resulted in no effects on male reproductive performance and no drug related effects in their offspring.

Perinatal and Postnatal Reproduction Study:

Four groups, each of 25 female rats with evidence of mating were administered 0, 1.8, 4.8, or 9.0 mg/kg/day of ketorolac tromethamine once daily by gavage from day 15 of pregnancy until 21 days postpartum or until all of their pups died. Females that did not litter were treated until approximately 25 days following the last day of mating and then sacrificed for pregnancy determination. Pups found dead within the first four days after parturition received an external examination and a skeletal examination if possible.

Ketorolac tromethamine at a dose of 9.0 mg/kg/day increased the length of gestation, the number of dams found dead or killed for cause as a result of dystocia, the number of pups found dead at first observation and, the number of pups dying within the first seven days postpartum. The weight of male and female pups was also decreased at days 4 and 7 postpartum compared to the control group.

Ketorolac tromethamine at a dose of 4.8 mg/kg/day did not alter the length of gestation of dams littering normally but did increase the incidence of dams found dead or sacrificed for cause as a result of dystocia. The maternal effects observed at the two highest dose levels were expected for a drug of this class.

Ketorolac tromethamine at a dose of 1.8 mg/kg/day caused no alterations in the length of gestation, nature of parturition, pup survival or any other aspect of reproductive performance.

Teratology:

Studies were conducted in rats and rabbits. Female rats (25 per group) were administered ketorolac tromethamine at doses of 0 (vehicle control), 0.1, 0.6 or 3.6 mg/kg/day by gavage, once daily from day 6 through day 15 of gestation.

At these doses no maternal toxicity or fetal anatomical abnormalities related to the administration of ketorolac tromethamine were observed.

In a second study, female rats which were administered ketorolac tromethamine 10 mg/kg orally by gavage once daily showed pallor, rough coat and lower body weight gains than the control dams. One dam died on gestation day 15; duodenal ulceration and peritonitis considered to be treatment related were seen. No embryotoxicity or embryolethality were observed. External and skeletal or visceral examinations of fetuses did not reveal any teratogenic changes attributable to the test compound.

Administration of ketorolac tromethamine, to female rabbits during organogenesis (day 6 through day 18 of gestation) by gavage once daily at doses of 0.1, 0.6 or 3.6 mg/kg/day was not teratogenic.

There were no treatment related clinical changes during the course of the study. One mid dose animal died on gestation day 18 of undetermined cause. All other animals survived to the end of the study. A slight body weight loss was noted in the high dose animals and there was a slight dose related reduction in food consumption during days 6 through 11 of gestation.

There were no statistically significant or biologically meaningful differences in the number of litters with malformations in any of the treated groups when compared to the control group. Developmental and genetic variations in fetuses were comparable for all groups.

REFERENCES

1. Anonymous. Ketorolac, KETOROLAC TROMETHAMINE INJECTION. Drugs of the Future. J.R. Prous S.A. Publishers (1989); 14:1010-1012.
2. Bloomfield SS, Mitchell J, Cissell GB, Barden TP and Yee JP.Ketorolac versus aspirin for postpartum uterine pain. Pharmacotherapy (1986); 6:247-252.
3. Bravo BLJC, Mattie H, Spierdijk J, Bovill JG, and Burm AGL. The effects on ventilation of ketorolac in comparison with morphine. Eur. J. Clin Pharmacol. (1988); 35(5): 491-494.
4. Buckley MM-T, Brogden RN. Ketorolac: A review of its pharmacodynamic and pharmacokinetic properties, and therapeutic potential. Drugs (1990); 39:86-109.
5. Conrad KA, Fagan TC, Mackie MJ and Mayshar PV. Effects of ketorolac tromethamine or hemostasis in volunteers. Clin. Pharmacol. Ther (1988); 43(5):542-546.
6. Estenne B, Julien M, Charleux H, Arsac M, Arvis G and Loygue J. Comparison of ketorolac, pentazocine and placebo in treating postoperative pain. Curr. Ther. Res. (1988); 43(6): 1173-1182.
7. Gannon R. Focus on ketorolac: a non steroidal, anti-inflammatory agent for the treatment of moderate to severe pain. Hosp. Formul. (1989); 24:695-702.
8. Gillies GWA, Kenny GNC, Bullingham RES and McArdle CS. Themorphine sparing effect of ketorolac tromethamine: A study of a new, parenteral non-steroidal anti-inflammatory agent after abdominal surgery. Anaesthesia (1987); 42:727-731.
9. Hillier K. BPPC. Drugs of the Future (1981); VI:669-670.
10. Honig WJ and Van Ochten, J. A multiple-dose comparison of ketorolac tromethamine with diflunisal and placebo in postmeniscectomy pain. J. Clin. Pharmacol. (1986); 26:700-705.
11. Johansson S, Josefsson G, Malstam J, Lindstrand A and Stenstroem A. Analgesic efficacy and safety comparison of ketorolac tromethamine and doleron for the alleviation of orthopaedic post-operative pain. The Journ. of Int. Med. Res. (1989); 17:324-332.
12. Jung D, Mroszczak E and Bynum L. Pharmacokinetics of ketorolac tromethamine in humans after intravenous, intramuscular and oral administration. Eur. J. Clin. Pharmacol. (1988); 35: 423-425.
13. Jung D, Mroszczak E, Wu A, Ling TL, Sevelius H and Bynum L. Pharmacokinetics of ketorolac and p-hydroxyketorolac following oral and intramuscular administration of ketorolac tromethamine. Pharmaceut. Res. (1989); 6:62-65.
14. Kagi P. A multiple-dose comparison of oral ketorolac andpentazocine in the treatment of post-operative pain. Curr. Ther. Res. (1989); 45(6): 1049-1059.
15. MacDonald FC, Gough KJ, Nicoll AG and Dow RJ. Psychomotor effects of ketorolac in comparison with buprenorphine and diclofenac. Br. J. clin. Pharmac. (1989); 27:453-459.
16. McQuay HJ, Poppleton P, Carroll D, Summerfield, RJ, Bullingham RES and Moore RA. Ketorolac and acetaminophen for orthopedic postoperative pain. Clin. Pharmacol. Ther.

(1986); 39(1):89-93.

17. Mroszczak E, Lee FW, Combs D, Sarnquist FH, Huang B-L, Wu AT, Tokes LG, Maddox ML, and Cho DK. Ketorolac tromethamine absorption, distribution, metabolism, excretion and pharmacokinetics in animals and humans. Drug Metab. Dispos. (1987); 15: 618-626.
18. Muchowski JM, Unger SH, Ackrell J, Cheung P, Cooper GF, CookJ, Gallegra P, Halpern O, Koehler R, Kluge AF, Van Horn AR, Antonio Y, Carpio H, Franco F, Galeazzi E, Garcia I, Greenhouse R, Guzman A, Iriarte J, Leon A, Pena A, Perez V, Valdez D, Ackerman N, Ballaron SA, Murthy DVK, Rovito JR, Tomolonis AJ, Young JM and Rooks WH. Synthesis and antiinflammatory and analgesic activity of 5-aroyl-1, 2-dihydro-3H-pyrrolo (1, 2-a) pyrrole-1- carboxylic acids and related compounds. J. Med. Chem. (1985) 28:1037-1049.
19. O'Hara DA, Fragen RJ, Kinzer M, and Pembertson D. Ketorolactromethamine as compared with morphine sulfate for treatment of postoperative pain. Clin. Pharmacol. Ther.(1987); 41:556-561.
20. Rooks WH, Tomolonis AJ, Maloney PJ, Wallach MB and Schuler ME. (1982) The analgesic and anti-inflammatory profile of (+)-5-benzoyl-1,2-dihydro-3H-pyrrolo(1,2a)pyrrole-1-carboxylic acid (RS-37619). Agents and Actions. (1982); 12(5):684-690.
21. Rooks WH, Maloney PJ, Shott LD, Schuler ME, Sevelius H, Strosberg AM, Tanenbaum L, Tomolonis AJ, Wallach MB, Waterbury D and Yee JP. The analgesic and anti-inflammatory profile of ketorolac and its tromethamine salt. Drugs Exptl. Clin. Res. (1985); XI:479-492.
22. Vangen O, Doessland S and Lindbeck E. Comparative study of ketorolac and paracetamol/codeine in alleviating pain following gynaecological surgery. J. Int. Med. Res. (1988); 16:443-451.
23. Wischnik A, Manth SM, Lloyd J, Bullingham R and Thompson JS. The excretion of ketorolac tromethamine into breast milk after multiple oral dosing. Eur. J. Clin. Pharmacol. (1989); 36:521-524.
24. Yee JP, Koshiver JE, Allbon C, and Brown CR. Comparison of intramuscular ketorolac tromethamine and morphine sulfate for analgesia of pain after major surgery. Pharmacotherapy (1986); 6(5):253-261.
25. Information Letter, Health Protection Branch. Nonsteroidal Anti-inflammatory Drugs. DD-33; August 21, 1985.
26. TORADOL Product Monograph, Control Number: 180668, Date of Revision: February 19, 2015.

PART III: CONSUMER INFORMATION

PrKETOROLAC TROMETHAMINE INJECTION, USP
ketorolac tromethamine injection

Read this information each time you refill your prescription in case new information has been added.

This leaflet is part III of a three-part "Product Monograph" published when ketorolac tromethamine was approved for sale in Canada and is designed specifically for Consumers. This leaflet is a summary designed specifically for you. It will not tell you everything about ketorolac tromethamine. See your health care provider and pharmacist regularly and ask them questions about your health and any medications you take.

ABOUT THIS MEDICATION

What the medication is used for:

Ketorolac Tromethamine Injection
Your health care provider has prescribed Ketorolac Tromethamine Injection for you for one or more of the following medical conditions:

- for short-term use in the hospital for pain relief after surgery (not to exceed 2 days)

What it does:
Ketorolac tromethamine, as a non-steroidal anti- inflammatory drug (NSAID), can reduce the chemicals produced by your body which cause pain and swelling.
Ketorolac tromethamine, as a non-steroidal anti-inflammatory drug (NSAID), does NOT cure your illness or prevent it from getting worse.
Ketorolac tromethamine only can relieve pain and reduce swelling as long as you continue to take it.

When it should not be used:
DO NOT TAKE KETOROLAC TROMETHAMINE if you have any of the following medical conditions:

- **have had or are planning to have heart bypass surgery**
- **have severe, uncontrolled heart failure**
- **have bleeding in the brain or other bleeding disorders**
- **are at 28 weeks of pregnancy or more**
- **are in labour and delivery**
- **are breastfeeding or planning to breastfeed**
- **are allergic to ASA (Acetylsalicylic Acid), other NSAIDs (Non-steroidal Anti-Inflammatory Drugs), Ketorolac Tromethamine Injection or its ingredients**
- **have active ulcer or bleeding from the stomach or gut have inflammatory bowel disease (Crohn's Disease or Ulcerative Colitis)**
- **have liver or kidney disease**
- **have high potassium in the blood**
- **are currently using probenecid or oxpentifylline**
- **are having central nervous system injection e.g. epidural, brain or spinal administration**
- **are going into have any major surgery**
- **are using other NSAIDs**

Patients who took a drug in the same class as ketorolac tromethamine after a type of heart surgery (coronary artery bypass grafting (CABG)) were more likely to have heart attacks, strokes, blood clots in the leg(s) or lung(s), and infections or other complications than those who did NOT take that drug.

Ketorolac tromethamine should NOT be used in patients under 18 years of age since the safety and effectiveness have NOT been established.

What the medicinal ingredient is:
ketorolac tromethamine

What the non-medicinal ingredients are:

Ketorolac Tromethamine Injection: 10% (w/v) alcohol, sodium chloride, sodium hydroxide, hydrochloric acid.

What dosage forms it comes in:
Ketorolac tromethamine is available as a solution for intramuscular injection (30 mg/mL).

WARNINGS AND PRECAUTIONS

If you have, or previously had, any of the following medical conditions, see your health care provider to discuss treatment options other than ketorolac tromethamine:

- **Heart Attack or Angina**
- **Stroke or Mini-stroke**
- **Loss of Vision**
- **Current Pregnancy (less than 28 weeks)**
- **Congestive Heart Failure**

Before taking this medication, tell your health care provider if you have any of the following:

- High blood pressure
- High cholesterol
- Diabetes mellitus or on a low sugar diet
- Thickening or hardening of your artery walls (Atherosclerosis)
- Poor circulation to your extremities

- Smoker or ex-smoker
- Kidney disease or urine problems
- Previous ulcer or bleeding from the stomach or gut
- Previous bleeding in the brain
- Bleeding problems
- Liver, biliary, pancreatic or renal problems
- Family history of allergy to NSAIDs, such as acetylsalicylic acid (ASA), celecoxib, diclofenac, diflunisal, etodolac, fenoprofen, flurbiprofen, ibuprofen, indomethacin, ketoprofen, ketorolac, mefenamic acid, meloxicam, nabumetone, naproxen, oxaprozin, piroxicam, rofecoxib, sulindac, tenoxicam, tiaprofenic acid, tolmetin, or valdecoxib (NOT a complete list)
- Family history of asthma, nasal polyps, long-term swelling of the sinus (chronic sinusitis) or hives
- Family history of allergy to sulfonamide drugs *(if applicable)*
- Any other medical problem

Also, before taking this medication, tell your health care provider if you are planning to get pregnant.

While taking this medication:

- tell any other doctor, dentist, pharmacist or other health care professional that you see, that you are taking this medication, especially if you are planning to have heart surgery;
- do NOT drink alcoholic beverages while taking this medication because you would be more likely to develop stomach problems;
- fertility may be decreased. The use of ketorolac tromethamine is not recommended in women trying to get pregnant. In women who have difficulty conceiving, stopping ketorolac tromethamine should be considered.

INTERACTIONS WITH THIS MEDICATION

Talk to your health care provider and pharmacist if you are taking any other medication (prescription or non-prescription) such as any of the following (NOT a complete list):

- Acetylsalicylic Acid (ASA) or other NSAIDs
  - e.g. ASA, celecoxib, diclofenac, ibuprofen, indomethacin, ketorolac, meloxicam, naproxen)
- Antacids
- Antidepressants
  - Selective Serotonin Reuptake Inhibitors (SSRIs)
    - e.g. citalopram, fluoxetine, paroxetine, sertraline
- Blood pressure medications
  - ACE (angiotensin converting enzyme) inhibitors
    - e.g. enalapril, lisinopril, perindopril, ramipril
  - ARBs (angiotensin II receptor blockers)
    - e.g. candesartan, irbesartan, losartan, valsartan
- Blood thinners
  - e.g. warfarin, ASA, clopidogrel
- Corticosteroids (including glucocorticoids)
  - e.g. prednisone
- Cyclosporin
- Digoxin
- Diuretics
  - e.g. furosemide, hydrochlorothiazide
- Lithium
- Methotrexate
- Morphine
- Oxpentifylline
- Probenacid

Your health care provider may prescribe low dose ASA (acetylsalicylic acid) as a blood thinner to reduce your risk of having a heart attack or stroke while you are taking ketorolac tromethamine. Take only the amount of ASA prescribed by your health care provider. You are more likely to upset or damage your stomach if you take both ketorolac tromethamine and ASA than if you took ketorolac tromethamine alone.

PROPER USE OF THIS MEDICATION

Ketorolac Tromethamine Injection

Usual dose (18-65 years of age):

Medical Condition	Starting Dose	Maximum Dose (per day)	Maximum Duration of Treatment (days)
Post-surgical or Musculo-skeletal Pain	Initial dose: 10 mg to 30 mg according to pain. Subsequent doses: 10 mg to 30 mg every 4 to 6 hours as needed to control pain	Doses exceeding 120 mg per day are not recommended	2 days

Administer ketorolac tromethamine only as directed by your health care provider. **Do NOT administer more of it, do NOT administer it more often and do NOT administer it for a longer period of time than your health care provider recommended. If possible, you should administer the lowest dose of this medication for the shortest time period.**

Administering too much ketorolac tromethamine may increase your chances of unwanted and sometimes dangerous side effects, especially if you are elderly, have other diseases or take other medications.

If a longer period is considered, see your health care provider regularly to discuss whether this medicine is working for you and if it is causing you any unwanted effects.

This medication has been prescribed specifically for you. Do NOT give it to anyone else. It may harm them, even if their symptoms seem to be similar to yours.

Ketorolac tromethamine is NOT recommended for use in patients under 18 years of age since safety and effectiveness have NOT been established.

Missed Dose:

The missed dose should be administered as soon as remembered, and then the regular dosing schedule should be continued. Two doses of ketorolac tromethamine should not be administered at the same time.

Overdose:

In case of drug overdose contact a health care practitioner, hospital emergency department, or regional Poison Control Centre immediately even if there are no symptoms.

SIDE EFFECTS AND WHAT TO DO ABOUT THEM

Ketorolac tromethamine may cause some side effects, especially when used for a long time or in large doses. When these side effects occur, you may require medical attention. Report all symptoms or side effects to your health care provider.

Ketorolac tromethamine may cause you to become drowsy or tired. Be careful about driving or participating in activities that require you to be alert. If you become drowsy, dizzy or light-headed after taking ketorolac tromethamine, do NOT drive or operate machinery.

Ketorolac tromethamine may cause you to become more sensitive to sunlight. Any exposure to sunlight or sunlamps may cause sunburn, skin blisters, skin rash, redness, itching or discolouration, or vision changes. If you have a reaction from the sun, check with your health care provider.

Check with your health care provider IMMEDIATELY if you develop chills, fever, muscle aches or pains, or other flu-like symptoms, especially if they occur before or together with a skin rash. These symptoms may be the first signs of a SERIOUS ALLERGIC REACTION to this medication.

SERIOUS SIDE EFFECTS AND WHAT TO DO ABOUT THEM		
**Symptom**	**STOP taking Ketorolac Tromethamine and get emergency medical attention IMMEDIATELY**	**STOP taking Ketorolac Tromethamine and talk to your physician or pharmacist**
Bloody or black tarry stools	✓	
Shortness of breath, wheezing, any trouble breathing or chest tightness	✓	
Skin rash, hives, swelling or itching	✓	
Blurred vision, or any visual disturbance	✓	
Any change in the amount or colour of your urine (red or brown)	✓	
Any pain or difficulty experienced while urinating		✓
Swelling of the feet, lower legs, weight gain		✓
Vomiting or persistent indigestion, nausea, stomach pain or diarrhea		✓
Yellow discolouration of the skin or eyes, with or without itchy skin		✓
Malaise, fatigue, loss of appetite		✓
Headaches, stiff neck		✓
Mental confusion, depression		✓
Dizziness, lightheadedness		✓
Hearing problems		✓

This is not a complete list of side effects. If you develop any other symptoms while taking ketorolac tromethamine , see your health care provider.

HOW TO STORE IT

Store ketorolac tromethamine at room temperature (15-30° C) away from light.

Do NOT keep outdated medicine or medicine no longer needed. Any outdated or unused medicine should be returned to your pharmacist

Keep out of sight and reach of children.

REPORTING SUSPECTED SIDE EFFECTS

You can report any suspected adverse reactions associated with the use of health products to the Canada Vigilance Program by one of the following 3 ways:

--

- **Report online at www.healthcanada.gc.ca/medeffect**
- **Call toll-free at 1-866-234-2345**
- **Complete a Canada Vigilance Reporting Form and:**
  - Fax toll-free to 1-866-678-6789, or
  - Mail to: Canada Vigilance Program
  Health Canada
  Postal Locator 0701E
  Ottawa, Ontario
  K1A 0K9

Postage paid labels, Canada Vigilance Reporting Form and the adverse reaction reporting guidelines are available on the MedEffect™ Canada Web site at www.healthcanada.gc.ca/medeffect.

NOTE: Should you require information related to the management of side effects, contact your health professional. The Canada Vigilance Program does not provide medical advice.

MORE INFORMATION

This document plus the full product monograph, prepared for health professionals can be found by contacting the sponsor, Juno Pharmaceuticals Corp. at: 1-855-819-0505

This leaflet was prepared by Juno Pharmaceuticals Corp.

Juno Pharmaceuticals Corp.
402-2233 Argentia Rd.
Mississauga, Ontario
L5N 2X7

Last Revised: September 21, 2020

All other trade-marks are the property of their respective owners.
