# Supplementary material for: Comparison of Ketorolac at 3 Doses in Children With Acute Pain: Protocol for A Randomized Controlled Trial
Source: JMIR Res Protoc. 2025 Sep 26;14:e76554. doi: 10.2196/76554 (PMC12550451; doi:10.2196/76554)
Supplement: Multimedia Appendix 3 [file resprot_v14i1e76554_app3.docx]

Assent

**Title: Comparison of Ketorolac at Three Doses in Children with Acute Pain: A Randomized Controlled Trial**

**Principal Investigator: Mohamed Eltorki, MBChB FRCPC**

Address: McMaster Children’s Hospital, Pediatric Emergency Medicine

1200 Main Street West, Hamilton, Ontario, L8S 4K1

Telephone: 905-521-2100 ext.76472

Email: [eltorkim@mcmaster.ca](mailto:eltorkim@mcmaster.ca)

**Co-Principal Investigator: Lucia Giglia, MD FRCPC**

Address: McMaster Children’s Hospital, Pediatric Emergency Medicine

1200 Main Street West, Hamilton, Ontario, L8S 4K1

Telephone: 905-525-9140 ext.73584

Email: [giglial@mcmaster.ca](mailto:giglial@mcmaster.ca)

**Co-Investigators:**

Samina Ali, MD, Department of Pediatrics, University of Alberta

Karen Beattie, BSc, PhD, Family Advisory Council, McMaster Children’s Hospital

Anne Holbrooke, MD, PharmD, FRCPC, St Joseph’s Healthcare Hamilton

Michael Livingstone, Department of Surgery, McMaster University

Myla Novak, Youth Advisory Council, McMaster Children’s Hospital

Samira Samiee-Zafarghandy, MD, Department of Pediatrics, McMaster Children’s Hospital

Lehane Thabane, PhD, McMaster University, Hamilton, Ontario

**Funding Source: Core Builder’s Grant, Department of Pediatrics, McMaster University**

**Why are we doing this study?**

Sometimes children may have bad headaches or tummy pain. This can be caused by migraine headaches or problems in the tummy like appendicitis or a stone in the kidney or gall bladder. This can make the tummy very sore and can even make children feel sick and throw up. Sometimes children can’t eat or drink because of their tummy or head pain. Children can take medicine to help the pain. There are many types of medicines that doctors can give children to help with their pain. The medicine used in this study is called ketorolac. This will be given to you to treat your pain, regardless of your participation in this study. Our study is comparing different amount of that medicine works best.

**Why am I being asked to be in the study?**

We are asking you to be in the study because you have bad pain (for example, headache, tummy ache) that needs to be treated with ketorolac. The advantage of using ketorolac, is that it can help really bad pain, it works quickly, and it does not require you to swallow it and can be given directly in your vein. We are trying to learn more about the best amount of this medicine to give. So, you will receive either the amount that we usually use, or two other amounts that are less than what we usually give. You will not know which amount of medicine you got.

**If I am in the study, what will happen to me?**

A nurse or a doctor will give you medicine through a plastic straw that is inserted in your vein by your nurses. Then we will come back and check on you a few times to ask how sore your tummy is. We will ask: “From 0 to 10, where 0 means no pain and 10 means the worst pain, how much pain do you have right now?”

**What if I have questions?**

You can ask the doctor any questions you have about this study. Even if you have questions later, you can call or email the doctor. His phone number is 905-521-2100 x76472 and his email is eltorkim@mcmaster.ca.

**Will I be hurt if I am in the study?**

We will give you pain medicine through your needle. The doctor who is treating you will decide if you need more pain medicine. Even if you were not in the study, you would still get the needle. This is because it’s the best way to give you pain medicine. Being in the study will not mean you get any more needles.

**Will the study help me?**

You will have more people looking after you and checking that your pain is getting better. Also, you will be helping other kids that come to the hospital because we will know more about how to treat their pain. You/caregiver will receive a small gift from us as a thank you for participating in our study.

**Do I have to be in this study?**

You do not have to be in this study if you do not want to be. If you decide that you don’t want to be in this study, it is OK. Nobody will be angry or upset. We also talked to your parent/caregiver about this study, and you should talk to them too.

**What happens after the study?**

When we are all finished with the study, we will write a report about what we learned. This report will not have your name or say that you were in this study. If you decide you want to be in this study, please print/write your name. You will receive a signed copy of this consent form.

I understand the study explained to me, all my questions have been answered and agree to participate.

________________________ _______________________ _______________

*(print name of participant) (Initials of participant) (date yyyy/mm/dd)*

I have explained to the participant all aspects pertaining to this study and I have answered the questions that they have asked me. I have indicated to them that participation in the study is voluntary and that they may withdraw at any time.

__________________________ __________________________ _______________

*(print name of person obtaining consent) (signature of person obtaining consent) (date yyyy/mm/dd)*

**Informed Consent Form for Participation in a Research Study participant or parent/caregiver)**

**Title: Comparison of Ketorolac at Three Doses in Children with Acute Pain: A Randomized Controlled Trial**

**Principal Investigators: Mohamed Eltorki, MBChB FRCPC**

Address: McMaster Children’s Hospital, Pediatric Emergency Medicine

1200 Main Street West, Hamilton, Ontario, L8S 4K1

Telephone: 905-521-2100 ext.76472

Email: [eltorkim@mcmaster.ca](mailto:eltorkim@mcmaster.ca)

**Lucia Giglia, MD FRCPC**

Address: McMaster Children’s Hospital, Pediatric Emergency Medicine

1200 Main Street West, Hamilton, Ontario, L8S 4K1

Telephone: 905-525-9140 ext.73584

Email: [giglial@mcmaster.ca](mailto:giglial@mcmaster.ca)

**Co-Investigators:**

Samina Ali, MD, Department of Pediatrics, University of Alberta

Karen Beattie, BSc, PhD, Family Advisory Council, McMaster Children’s Hospital

Anne Holbrooke, MD, PharmD, FRCPC, St Joseph’s Healthcare Hamilton

Michael Livingstone, Department of Surgery, McMaster University

Myla Novak, Youth Advisory Council, McMaster Children’s Hospital

Samira Samiee-Zafarghandy, MD, Department of Pediatrics, McMaster Children’s Hospital

Lehane Thabane, PhD, McMaster University, Hamilton, Ontario

**Funding Source: Core Builder’s Grant, Department of Pediatrics, McMaster University**

*As a Substitute Decision Maker, you are being asked to provide informed consent on behalf of a person who is unable to provide consent for themselves. If the participant gains the capacity to consent for him/herself, your consent for them will end.* *Throughout this form, “you” means the person you are representing.*

You are being invited to participate in a research study because you have moderate to severe pain and will be receiving a pain medication called ketorolac. This is the only non-opioid pain medicine given through the intravenous line. It is used for treating pain from conditions such as migraine, suspected appendicitis or kidney or bladder stones or ovarian cysts. It is particularly useful when children are in moderate to severe pain, and they can’t eat or drink because their tummy is upset, or they are awaiting results of tests. This form gives you information about the study. Someone will talk to you about the study so you can decide if you want to be in it. Please read this form carefully and ask any questions you may have. Make sure you have all your questions answered before you decide if you want to be a part of the study. It is important for you to know that you can choose not to take part in this study. The appropriate care you will get at the hospital will not be affected if you choose not to be part of it. 171 participants will be in this trial.

**Why is this research study being done?**

Ketorolac is a pain medication that belongs to a class of pain killers called non-steroidal anti-inflammatory drugs (NSAIDs). A common example of that given by mouth is ibuprofen. Ketorolac is very effective in treating pain and is particularly useful for moderate to severe pain when oral medications are either ineffective or cannot be given due to tummy upset (vomiting is common with migraine or severe abdominal pain) or children are instructed not to eat or drink in case they need an operation. It is the only non-opioid we have available to be given through a needle called an intravenous (IV).

In our emergency department, we use ketorolac for painful problems like kidney and gall bladder stones, migraine headaches, appendicitis, pains from a period, ovarian cysts, burns and pain from an injury. We know from a few studies that it might be just as good as morphine to help with pain. Even though we know ketorolac well, we do not have good studies that can tell us about the best dose to give. Studies from adult patients, show that a low dose of ketorolac maybe just as good as standard dose. However, we cannot take that at face-value and apply it to children since the dosing in children is quite different and they respond and breakdown medicines very differently than adults.

The goal of this research is to see if low dose ketorolac works just as well as standard dose ketorolac. We also need to know if children who get low-dose ketorolac have the same or less side effects than children who get standard dose ketorolac. To answer this research question, we will need many patients in a study.

**What will happen during this research study**?

If you decide to be in this study, you will receive pain medicine through the intravenous. Several measures are in place to avoid delay in administering the pain medicine. You will get one of three following doses:

1. Ketorolac given at 0.5 mg/kg to a maximum of 30 mg OR
2. Ketorolac given at 0.5 mg/kg up to a maximum of 10 mg OR
3. Ketorolac given at 0.25 mg/kg to a maximum of 30 mg.

The decision on which one you will get will be random, just like flipping a coin. In addition to ketorolac, you will get two small doses of salt water, called saline. This to make sure no one can guess what dose ketorolac you received. No one will know which dose ketorolac you will get except the hospital pharmacist. You, doctors, nurses, and research staff will not know which ketorolac dose you are getting, unless the doctor needs to know for special reasons (example: you had a rare or unexpected reaction). At the end of the study, after 171 children have been in it, the doctor in charge of the study will find out which medicine each child received.

In order to know if the pain medicine is working, we will measure your pain. This is done by asking you/your child “On a scale of 0 to 10, where 0 means no pain and 10 means the worst pain, how much pain do you have right now?” We will ask this question a few times while you/your child are getting the medicine. Then, after all the pain assessments are completed, we will ask you/your child to complete a survey in-person if you are still at the hospital or by phone or email within 7 to 14-days. The survey is to understand your/your child perception of pain, pain medicine and side effects associated with them.

**What are the risks/benefits to doing this research study?**

While prior evidence from studies done on adult participants show that low-dose ketorolac is not any worse than standard dose without any difference in side effects, there is a risk that in children this is not the case and that our participants in the low-dose arm will have inferior pain control. To avoid any poor pain control, we have allowed other pain medicine to be administered as needed by your doctor and nurse. Intravenous ketorolac, common side effects may include nausea, upset stomach, itching, or rash or pain at the IV site. Rarely or with prolonged usage (after several days to weeks), you may get a stomach ulcer or bleeding or an allergic reaction. Rarely, you may experience an allergic reaction or difficulty breathing. All children who participate are allowed to take acetaminophen (Tylenol) if they need to. If you have bad pain after you get the medicine, your bedside clinical team can give you more pain medicine (morphine). You will receive appropriate attention during the study.

**How will I be compensated?**

If you choose to participate, we will thank you by giving you a gift card at the end of the study.

**How can I withdraw from the research study?**

If you wish to stop being in the study, you can call the study researchers at any time: Dr. Mohamed Eltorki (905 521 2100 extension 76472 or at [eltorkim@mcmaster.ca](mailto:eltorkim@mcmaster.ca)), or the study coordinator Redjana Carciumaru (905-521-2100 extension 73864 or at carciur@mcmaster.ca).

**How will the information we collect be kept private?**

All the information you give us will be confidential (private). Only the study’s research team will be seeing the information. The information will be stored on a computer that will not have your name or any other information that may be used to identify you. There is a risk of breach of confidentiality related to participation in the study. Several measures are in place to keep all medical information that we collect confidential. Following completion of the study, data will be kept in a secure location for a minimum of 15 years as per the requirements of regulatory authorities.

Your participation in this study will be recorded in your electronic health record (EHR), also called a medical record, at Hamilton Health Sciences. If you participate, some of the information about you that is collected for this study, including the results of tests described in this consent form, will be stored in your EHR and accessible to others working at this hospital (like your current and future health care provider(s)). This hospital may share patient information stored in its EHR with other hospitals and healthcare providers in Ontario. In addition, any person or company to whom you give access to your medical record may have access to this information. The study team can tell you what information about you will be stored electronically, and what may be shared outside of this hospital.

**How do I find out what was learned in the research study?**

If you decide to be in the study, we would be happy to provide you a copy of the results.

**What happens if I have a research-related injury?**

There is very small risk of injury from being in this research study. If you suffer an injury from participation in this study, medical care will be made available to you by your study doctor, or you will be referred for appropriate medical care. However, if you sign this consent form it does not mean that you waive any legal rights you may have under the law, nor does it mean that you are releasing the investigators from their legal and professional responsibilities.

For the purposes of ensuring the proper monitoring of the research study, it is possible that a member of any institutional regulatory body, the Research Ethics Board, and a Health Canada representative may consult your research data and medical records. However, no records which identify you by name or initials will be allowed to leave the hospital. By signing this consent form, you authorize such access. This study has been reviewed by the Hamilton Integrated Research Ethics Board (HIREB). The HIREB is responsible for ensuring that participants are informed of the risks associated with the research, and that participants are free to decide if participation is right for them. If you have any questions about your rights as a research participant, please call the Office of the Chair, Hamilton Integrated Research Ethics Board at 905.521.2100 x 42013.

**Consent:**

I understand the nature of the project explained to me, including all the potential benefits and risks, hereby agree to participate in the study. I will receive a signed copy of this consent form.

________________________ _______________________ _______________

*(print name of participant)(if applicable) (signature of participant) (date yyyy/mm/dd)*

__________________________ __________________________ _______________

*(print name of Substitute Decision Maker) (signature of Substitute Decision Maker) (date yyyy/mm/dd)*

I have explained to the participant all aspects pertaining to this study and I have answered the questions that they have asked me. I have indicated to them that participation in the study is voluntary and that they may withdraw at any time.

The research project must be explained to the participant, and a member of the research team must answer their questions, specifying that participation in the project is free and voluntary. The research team will respect the terms of the consent form.

__________________________ __________________________ _______________

*(print name of person obtaining consent) (signature of person obtaining consent) (date yyyy/mm/dd)*
