# Supplementary material for: Comparison of Ketorolac at 3 Doses in Children With Acute Pain: Protocol for A Randomized Controlled Trial
Source: JMIR Res Protoc. 2025 Sep 26;14:e76554. doi: 10.2196/76554 (PMC12550451; doi:10.2196/76554)
Supplement: Multimedia Appendix 4 [file resprot_v14i1e76554_app4.pdf]

Follow-Up Survey (7 to 14 days post ED discharge)

Call must be done in this time window

7 days from discharge: Date:__________/_________________/___________(dd/mmm/yyyy)

14 days from discharge: Date:__________/_________________/___________(dd/mmm/yyyy)

**Follow-up Call Attempts:**			
Number of call attempts made (circle): 1 2 3 N/A – completed via email			
	**Date and Time** *(dd/ mmm/ yyyy HH:MM)*	**RA Initials**	**Comments**
**Call # 1:**	____/____/______ ____:_____		
**Call # 2:**	____/____/______ ____:_____		
**Call # 3:**	____/____/______ ____:_____		

Section A: Knowledge of Analgesia

Please answer the following questions on a scale of 1 to 7, 1 is not at all effective and 7 is very effective.							
	1	2	3	4	5	6	7
In your experience, how effective is acetaminophen (Tylenol) in treating your child's pain?	☒	☐	☐	☐	☐	☐	☐
In your experience, how effective is ibuprofen (Advil or Motrin) in treating your	☐	☐	☐	☐	☐	☐	☐

child's pain?							
In your experience, how effective is acetaminophen (Tylenol) AND ibuprofen (Advil, Motrin) when given together or alternating them, in treating your child's pain	☐	☐	☐	☐	☐	☐	☐
In your experience, do you think prescription pain medicine works better than over the counter medicine	☐	☐	☐	☐	☐	☐	☐
In your experience, do you think pain medicine given at the hospital works better than over the counter medicine? Where 1 is 0% more effective than over the counter medication and 7 is at least a 100% more effective than over the counter medication.	☐	☐	☐	☐	☐	☐	☐
Do you think pain medication given through a vein (requires a needle poke) is more effective than pain medicine taken by mouth? Where 1 is 0% better than oral	☐	☐	☐	☐	☐	☐	☐

medicine and 7 is at least 100% better than oral medicine.							
On a scale of 1 to 7, to what extent do you agree with this statement:<br>"When I bring my child to the hospital, I expect his or her pain to be treated by something else other than over the counter medication or what I have given at home? Where 1 is 0% agreement and 7 is 100% agreement.	☐	☐	☐	☐	☐	☐	☐
Other than ibuprofen (Advil, Motrin), acetaminophen (Tylenol), can you name any other types of pain medicines that you know about?	☐ Yes<br>If yes please name it:<br>1.<br>2.<br>3.<br>4.<br>5.					☐ No	
Next Section							
When do you feel it is best to give pain medicine?<br>Please choose the most appropriate answer	A) As soon as the pain starts<br>B) Only if the pain reaches middle level<br>C) When the pain is at its worst						

On a scale of 1 to 7, if the pain completely goes away, how likely would you be to continue pain medicine as instructed by the health care provider for the entire period recommended (e.g., 1 week of continuous pain medicine given every 4-6 hours).<br>Where 1 is not at all likely and 7 is very likely.	☐	☐	☐	☐	☐	☐	☐

Section B: IV Analgesia

Other than vaccines (e.g. flu shots or COVID shots given at your doctor's office), have you ever needed medications where a needle was used?<br>(Preferred to ask the child or adolescent)	☐ Yes	☐ No
On a scale of 0 to 10 where 0 is not scared at all and 10 is extremely scared how scared (anxious) are you about getting a needle for medications of for blood tests?	________/10	
On a scale of 0 to 10 where 0 is not at all painful and 10 is extremely painful, how painful was the actual procedure of getting a needle for a medication or blood test?	________/10	
If you came in with the same pain again, would you want the same treatment given?	☐ Yes	☐ No<br>If No, would you rather have the treatment given by (Check all that apply):<br>A) Mouth<br>B) Intranasal spray

		C) Intramuscular shot (injection in the muscle, like a vaccine)<br>D) Subcutaneous shot (injection in the skin, like a vaccine but hurts a bit less)
What was worse, the pain from having an intravenous put in using a needle or the pain you had from your condition?	A) Intravenous put in using a needle<br>B) Underlying painful condition	
On a scale of 1 to 7 where 1 is not at all worth it and 7 is extremely worth it, was the pain relief that you had from the medicine given by an intravenous that was put in using a needle worth it?	_______/7	

Section C: Analgesia Dosing and Adverse Events (Questions to caregivers):

If your child is in a lot of pain and higher doses of pain medicine might help more for your child's pain, but might also cause more side effects (such as drowsiness or stomach ache), what amount of pain medicine would you prefer to start with your child:	☐ Low Dose<br>☐ Medium Dose<br>☐ High Dose	
If there was no difference in pain relief between low and higher doses of medication, but higher doses of medication might cause more side effects (such as drowsiness or stomach ache), what amount of medication would you prefer for your child:	☐ Low Dose<br>☐ Medium Dose<br>☐ High Dose	
Has your child ever had side effects form pain medication before?	☐ Yes<br>If Yes, what were those side effects?<br>1.<br>2.<br>3.<br>4.	☐ No
How worried are you about side effects form pain medication? 1 is not worried at all and 7 is extremely worried	_______/7	

22) Which side effects concern you more?

CHECK ALL THAT APPLY	SIDE-EFFECT	1 (not all worried)						7 (extremely worried)
☐	Allergic Reaction / Anaphylactic Shock							
☐	Constipation							
☐	Diarrhea							
☐	Drowsiness or Dizziness							
☐	Headache							
☐	Interaction with other medication							
☐	Nausea and or Vomiting							
☐	Substance abuse or addiction in the future							
☐	Stomach pain							
☐	Free text:							
☐	Free text:							

Do you have any other comments or concerns regarding this study?

Do you have any recommendations for us to improve our study design?

Thank you for completing this follow-up survey, we appreciate your participation in the KETODOSE study! Without families like you, our research would not be possible.
