# Supplementary material for: Comparison of Ketorolac at 3 Doses in Children With Acute Pain: Protocol for A Randomized Controlled Trial
Source: JMIR Res Protoc. 2025 Sep 26;14:e76554. doi: 10.2196/76554 (PMC12550451; doi:10.2196/76554)
Supplement: Multimedia Appendix 5 [file resprot_v14i1e76554_app5.pdf]

Date: Mar-21-2023

Local Principal Investigator: Dr. Mohamed Eltorki

Participating HiREB Centre(s): Hamilton Health Sciences

Project ID: 15587

Project Title: Comparison of Ketorolac at Three Doses in Children with Acute Pain: A Randomized Controlled Trial (KETODOSE TRIAL)

Review Type: Full Board

Meeting Date: Mar-01-2023

Date of Final Approval: Mar-21-2023

Ethics Expiry Date: Mar-21-2024

The Hamilton Integrated Research Ethics Board (HiREB) Panel B has reviewed and approved the abovementioned study.

The following documents have been approved:

Document Name	Document Date	Document Version
MCH KETODOSE Master Participant List V 1.0 7 Feb 2023	Feb-07-2023	V 1.0
1.7.1.1 Protocol_KETODOSE V 2.0 Feb 28 2023 Clean	Feb-28-2023	V 2.0
1.7.2.1 Ketodose Assent Form V 2.0 March 10 2023 Clean	Mar-10-2023	V 2.0
KETODOSE CRF V 1.0 March 9 2023 Clean	Mar-09-2023	V 1.0
ketorolac Product Monograph Template - Standard	Sep-21-2020	V 1.0
1.7.2.2 Ketodose ICF For Participation in a Research Study V 2.0 March 20 2023_CLEAN	Mar-20-2023	2.0
KETODOSE CRF V 2.0 March 20 2023 CLEAN	Mar-20-2023	2.0

The following documents have been acknowledged:

Document Name	Document Date	Document Version
HC Division 5 Jan 2023	Jan-24-2023	1
NOL270872	Jan-12-2023	1
Eltorki GCP 23 Jan 2023_ 23 Jan 2026	Jan-23-2023	V 1.0
CoRE Builder Team Grant - Budget Template.xlsx - Read-Only	Mar-18-2023	1

While HiREB has reviewed and approved this application, the research must be conducted in accordance with applicable regulations and institutional and/or public health requirements.

We are pleased to issue final approval for the above-named study until the expiry date noted above. Continuation beyond that date will require further review and renewal of HiREB approval. Any changes or revisions to the original submission must be submitted on a HiREB amendment form for review and approval by the Hamilton Integrated Research Ethics Board.

REB members involved in the research project do not participate in the review, discussion or decision.

The Hamilton Integrated Research Ethics Board (HiREB) provides ethical review and ongoing ethical oversight on behalf of Hamilton Health Sciences, St. Joseph's Healthcare Hamilton, Research St. Joseph's-Hamilton, the Faculty of Health Sciences at McMaster University and Niagara Health. HiREB operates in compliance with and is constituted in accordance with the requirements of: The Tri-Council Policy Statement on Ethical Conduct of Research Involving Humans (TCPS 2); The International Conference on Harmonisation of Good Clinical Practices Guideline (ICH GCP); Part C Division 5 of the Food and Drug Regulations, Part 4 of the Natural Health Products Regulations; Part 3 of the Medical Devices Regulations and the provisions of the Ontario Personal Health Information Protection Act 2004 and its applicable Regulations. For studies conducted at St. Joseph's Healthcare Hamilton, HiREB complies with the Health Ethics Guide of the Catholic Alliance of Canada. HiREB is qualified through the Clinical Trials Ontario (CTO) REB Qualification Program and is registered with the U.S. Department of Health and Human Services (DHHS) Office for Human Research Protection (OHRP).

Sincerely,

Frederick A Spencer

Dr. Frederick A. Spencer, MD
Chair, Hamilton Integrated Research Ethics Board

Hamilton Integrated Research Ethics Board (HiREB)
237 Barton Street, Suite C1-205 Hamilton, ON L8L 2X2
Telephone: 905-521-2100, Ext. 42013
