# Supplementary material for: Comparison of Ketorolac at 3 Doses in Children With Acute Pain: Protocol for A Randomized Controlled Trial
Source: JMIR Res Protoc. 2025 Sep 26;14:e76554. doi: 10.2196/76554 (PMC12550451; doi:10.2196/76554)
Supplement: Multimedia Appendix 7 [file resprot_v14i1e76554_app7.pdf]

McMaster University | Pediatrics

June 10, 2022

Dr Mohamed Eltorki
Assistant Professor
Department of Pediatrics
McMaster University

Dear Drs Eltorki & Giglia,

Thank you for submitting your project entitled "*Comparison of Ketorolac at three doses in children with acute pain: A randomized controlled trial (KETO-DOSE TRIAL)"* to the inaugural 2021/22 CoRE Builder Team Grants competition in the Department of Pediatrics. Through a yearly call for proposals, this initiative will support collaborative research excellence across our Department. We have been incredibly impressed by the rich teams and ideas put forward in eight projects this round.

On June 3, a review committee of four faculty members external to the Department of Pediatrics met to select projects for funding. We are thrilled to let you know that your team's project has been selected for funding! On the following pages, you will find reviewers comments, as well as feedback on your lay summary provided by a group of parent advisors. We encourage you to consider this feedback as you plan for project start-up and implementation. To open a new University research account for your funds, please complete an HRS Account Request form and submit to Melanie O'Brien obriem35@mcmaster.ca who will facilitate the process in Pediatrics and communicate your next steps.

We intend to use feedback from this competition to improve future competitions and would value your input to that end about what you liked, as well as any changes that would enhance the competition and/or applicant experience. Please take < 5 minutes to share your thoughts online – anonymously, if you wish.

We recognize and appreciate the time and effort taken to prepare your application, particularly amidst ongoing pandemic challenges. The collaborative spirit in our Department is vibrant – we look forward to continuing to grow and celebrate this important element of our research culture. If there is any way we can further support your team, do let us know.

Sincerely,

Katherine M. Morrison

Dr Katherine Morrison,
Associate Chair, Research
Department of Pediatrics
McMaster University

Review Discussion Overview

This is a clear and well-written grant that addressed all aspects of the proposed trial and potential challenges. Future directions are well-defined and address an important question in the pediatric population. Explicit linkage to Department of Pediatrics' MVV and additional clarity around positioning of the proposed study within the larger research program would have been helpful, as would comment around possible subgroup analyses for different pain etiologies.

Reviewer 1 Feedback

Study Description

This mixed methods study sets out to assess effective dosing of Keterolac, an opioid alternative for pain management in the pediatric population. This drug is currently used as an off-label drug and adult dosing regimes are employed because it is untested in the pediatric population. Due to pharmacokinetic differences among pediatric patients compared to adults, it is possible that higher doses may be required to effectively manage pain. The primary outcome of pain reduction is studied using a single-centre, block-randomized, double dummy, double blind, three-arm trial with parallel groups. A sample size of 60 per group will be recruited. The secondary objective will be addressed in parallel to the trial using semi-structured surveys, focus groups and through blood samples for future testing of ketorolac levels.

Strengths

This study will fill a much gap in knowledge regarding best dosing for Keterolac, an opioid alternative for management of pain in the pediatric population.

Reviewer 2 Feedback

Evidence-based pharmacological treatments for children with acute pain remain under-studied, and children are vulnerable to adverse events with pain medications regimens extrapolated from adult regimens. This proposal aims to improve the knowledge of safe and effective analgesics to treat children's acute pain by determining the most effective and safe dose of ketorolac for children with acute pain. It will also describe the experiences, attitudes, knowledge, and perspectives of the family unit in pain care and decision-making.

The primary research objective is to determine if in children aged 6-17 years with moderate to severe pain who are prescribed IV ketorolac by their treating physician, are lower-dose IV ketorolac dosing strategies non-inferior to standard dosing. They will also study patients' and caregivers' knowledge, attitudes, perceptions, and emotions on pain, medications, and their use in treating acute pain. Finally they will also examine the pharmacokinetics and pharmacodynamics (PK/PD) relation of ketorolac administered at standard and lower doses in children.

The program addresses a clear need in child health, and the rationale is strong with clearly outlined goals. It will generate preliminary data that could potentially support a future application for Tri-council funding that has strong likelihood of success. Methods are mixed and methodology is well detailed for clinical trial design. Issues of sex, gender, equity, diversity, & inclusion are not clearly addressed in the design, and could be better elucidated. The project is feasible and the knowledge translation activities are clearly defined and appropriate for the work proposed. Applicants have appropriate expertise to undertake this proposal, as their currently funded research program includes a comparative effectiveness network meta-analysis of all pharmacologic analgesia used in children with acute pain; a pilot Health Canada-approved single-centre randomized controlled trial (RCT) comparing the efficacy of

intravenous (IV) ketorolac to IV morphine for children with acute pain; and a quality improvement (QI) project aiming to improve provision of analgesia in children with acute abdominal pain.

Some limitations in study design may exist around confounding factors that may arise in treating multiple different sources of pain which may respond differentially to ketorolac; children with multiple pain sources will be admitted to the study and more methodological detail could describe whether subgroup analyses will be performed and whether metrics need to be adjusted for different etiologies of pain

Budget: Appropriate. No overlap.

CoRE Builder Team Grants

Parent Advisor Feedback

As part of the CoRE Builder Team Grants review process, parent advisors were invited to provide feedback on project lay abstracts.

A small group of parents of children cared for at McMaster Children's Hospital took keen interest in learning more about research in the Department of Pediatrics and generously shared their time and perspectives.

For each project, parents were asked to provide their general impressions of the project guided, in part, by questions including:

- Is the summary easy to understand?
- Are you convinced this study is important?
- If this was your own personal money to spend, would you fund it?
- If your child/you were eligible to participate, would you?
- What are the strengths of the application and the study? What are the weaknesses?
- What feedback/advice/questions do you have for the study team?

On the following page, you will find a summary of parents' feedback on your team's project organized by perceived strengths, weaknesses, questions, and suggestions.

STRENGTHS

- Very strong opening with the 2 scenarios - "could picture it in my mind"
- Well written - everything makes sense and importance is clear
- Appreciated the team's approach to learning more about patients' experiences via focus groups

WEAKNESSES

- Not immediately clear that 'Ketoralac' is the medication name

QUESTIONS

- How many children will participate in the study?

SUGGESTIONS

- Consider describing, in general terms, purpose of randomization, process of blinding, and who knows how much medication the child is receiving

CoRE Builder Team Grants

Parent Advisor Feedback
