# Supplementary material for: Comparison of Ketorolac at 3 Doses in Children With Acute Pain: Protocol for A Randomized Controlled Trial
Source: JMIR Res Protoc. 2025 Sep 26;14:e76554. doi: 10.2196/76554 (PMC12550451; doi:10.2196/76554)
Supplement: Multimedia Appendix 6 [file resprot_v14i1e76554_app6.pdf]

Health Canada | Santé Canada

Pharmaceutical Drugs Directorate
5th Floor, Holland Cross, Tower B
Address Locator # 3105A
OTTAWA, Ontario
K1A 0K9

12 January 2023

Dr. Mohamed Eltorki, MD
Hamilton Health Sciences Corporation
1200 Main Street West, 2R104
HAMILTON**,** Ontario
L8N 3Z5
(905) 521-2100 Ext. 76472
eltorkim@mcmaster.ca

Your file *Votre référence*
HC6-024-c270872
Our file *Notre référence*

No Objection Letter RE: Protocol # KETODOSE20221107

Dear Dr. Mohamed Eltorki:

I am pleased to inform you that the information and material to support your Clinical Trial Application for **KETOROLAC,** control number **270872**, received on 21 December, 2022 have been reviewed and we have no objection to your proposed study.

I would remind you of the necessity of complying with the *Food and Drug Regulations*, Division 5, in the sale of this product for clinical testing. In addition, the regulations impose record keeping responsibilities on those conducting clinical trials. You are also reminded that all clinical trials should be conducted in compliance with the Pharmaceutical Drugs Directorate 's *Guideline for Good Clinical Practice.*

You are reminded of the following requirements:

- Reports for all serious and unexpected Adverse Drug Reactions (ADRs) should be:
  - Faxed to 613-941-2121 (for therapeutics only). **OR**
  - Submitted electronically via the E2B Electronic Gateway. This method is recommended if your company/institution has electronic gateway capability. Please contact the Trading Partner Management Office (TPMO) by email at tpmo-bgpc@hc-sc.gc.ca for more information.

- All Clinical Trial Notifications should be emailed to oct.ctan-ndec.bec@hc-sc.gc.ca and formatted in accordance with Health Canada’s *Guidance Document: Preparation of Drug Regulatory Activities in the “Non-eCTD Electronic Only” Format.*

- A completed Clinical Trial Site Information Form for each Canadian site should be emailed to clinical.trials.site-lieu.essai.clinique@hc-sc.gc.ca prior to initiating the trial at that site.

Consistent with Health Canada's Notice - *Registration and Disclosure of Clinical Trial Information* of November 30, 2007, sponsors are encouraged to register their clinical trials within 21 days of the trial's onset, using a publicly available registry that conforms with international standards for registries such as:

- Clinicaltrials.gov (www.clinicaltrials.gov)
- Current Controlled Trials (www.controlled-trials.com)

Should you have any questions concerning this letter, please contact the Office of Clinical Trials (613) 941-2132.

Sincerely,

This document has been signed electronically using the Health Canada docuBridge system.

Canada

Larissa Lefebvre
Manager, Submission Management Division
Office of Clinical Trials

LL/kh
